# Adherence to healthy and sustainable diets and health-related behaviors in a Spanish online university setting

**DOI:** 10.3389/fnut.2026.1757733

**Published:** 2026-03-12

**Authors:** Maria Pilar Giner, Barbara Pilar González-Serrano, Alba Pardo, Alicia Aguilar-Martínez, Clara Gomez-Donoso, Paula Sol Ventura, Laura Esquius, Marina Bosque-Prous, Anna Bach-Faig

**Affiliations:** 1 NUTRALiSS, Faculty of Health Sciences, Open University of Catalonia (UOC), Barcelona, Spain; 2 Nestlé Research, Vers-chez-les Blanc, Lausanne, Switzerland; 3 High Performance and Health Applied Technology Research Group - (TAARS) TecnoCampus, Department of Health Sciences, Pompeu Fabra University, Mataró, Spain; 4 FoodLab Research Group, Faculty of Health Sciences, Open University of Catalonia (UOC), Barcelona, Spain; 5 Faculty of Health Sciences, Open University of Catalonia (UOC), Barcelona, Spain; 6 Department of Pediatrics, Hospital Universitari Arnau de Vilanova, Lleida, Spain; 7 Epi4health Research Group, Department of Psychobiology and Methodology of Health Sciences, Universitat Autònoma de Barcelona, Barcelona, Spain; 8 CIBER de Epidemiología y Salud Pública (CIBERESP), Madrid, Spain

**Keywords:** dietary pattern, emotional well-being, health promotion, healthy behaviors, online university, physical activity, university population

## Abstract

**Background:**

Universities represent strategic environments for promoting healthy behaviors. Evaluating dietary quality and sustainability alongside other health-related factors is essential for developing health promotion strategies.

**Objective:**

This study aimed to assess adherence to diets based on the latest Spanish healthy and sustainable dietary guidelines, and to explore associations between diet, health-related behaviors, and sociodemographic characteristics among students and staff at the Open University of Catalonia (UOC).

**Methods:**

An online cross-sectional survey was conducted during the 2020–2021 academic year (*n* = 2,608; 2,075 students, 533 staff). Data on food intake (via a semi-quantitative food frequency questionnaire), physical activity (PA), sedentary behavior (SB), sleep quality, and emotional well-being were collected. HEAlthy and SUStainable diet index (HEASUS) was computed using a continuous gradient-based scoring system for 18 food groups, ranging from −1 (lowest adherence) to 10 (highest adherence). Quantile regression models were employed to examine determinants of HEASUS adherence across tertiles, accounting for health-related and sociodemographic covariates.

**Results:**

Overall, many participants exhibited low PA, insufficient sleep, and elevated stress. Adherence to healthy and sustainable diet according to HEASUS was moderate (students: 5.9 ± 1.5; staff: 6.2 ± 1.4). Higher PA and active commuting were positively associated with HEASUS, while SB, male gender, and overweight status were inversely associated. Health-related factors showed stronger associations with HEASUS adherence at lower quantiles, indicating that unhealthy behaviors cluster and reinforce each other more strongly among those with poorer dietary adherence. Although females scored slightly higher on HEASUS, they reported greater stress, poorer sleep, and higher SB.

**Conclusion:**

Our findings underscore the importance of integrated, gender-sensitive strategies that jointly address diet quality, PA, sleep, and emotional well-being. Online universities represent an important setting for promoting both health and sustainability, especially through targeted actions that support a plant-rich, nutrient-dense dietary pattern, enhance emotional well-being, and encourage more active daily routines.

## Graphical abstract

**Figure:**
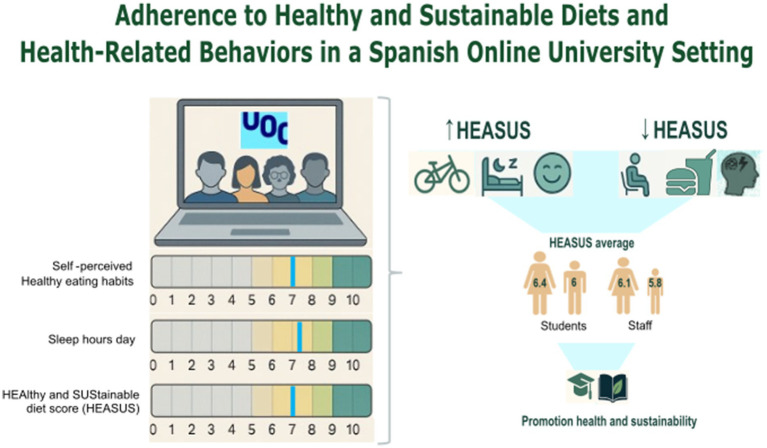

## Introduction

Non-communicable diseases (NCDs) remain one of the leading global health challenges, contributing substantially to morbidity and mortality worldwide (2). Modifiable lifestyle factors, including tobacco and alcohol use, physical inactivity, sedentary behavior, unhealthy diets, and insufficient sleep, are major drivers of this burden (3). Unhealthy dietary shifts not only contribute to the rising prevalence of NCDs but also to environmental degradation, underlying the intertwined nature of human and planetary health (4–7).

Universities represent strategic settings for health promotion, as they bring together students and staff in educational, occupational, and social contexts where long-lasting lifestyle habits are formed (8, 9). Universities function simultaneously as workplaces, educational institutions, and centers for knowledge transfer, placing them in a unique position to implement holistic health-promotion policies (10–12). The university community—including both students and staff—is crucial for fostering a supportive environment conducive to health. While most health promotion efforts focus primarily on students (13, 14), the well-being of university staff is equally important for building healthy campuses, particularly in virtual settings such as the Open University of Catalonia (UOC), a pioneer in online learning.

Evidence indicates that health behaviors can differ between online and traditional in-person university environments. During the COVID-19 pandemic, longer periods of online learning were linked to skipping breakfast and increased consumption of sugar-sweetened beverages (15, 16). While pandemic restrictions may have intensified these habits, similar lifestyle challenges persist in online and hybrid university contexts. Indeed, factors such as academic workload, stress, financial constraints, and easy access to inexpensive, nutrient-poor foods have historically contributed to unhealthy dietary and lifestyle behaviors in university populations, independent of pandemic conditions (17, 18). Sedentary behavior, emotional distress, and poor sleep often interact, leading to compensatory or disordered eating patterns (19, 20). Conversely, healthier behaviors—such as balanced diets, sufficient sleep, and regular physical activity—support better learning outcomes, emotional well-being, and productivity (21, 22). Together, these findings highlight the relevance of universities—particularly online institutions—as strategic settings for the promotion of healthy and sustainable lifestyles.

In response to these challenges, several initiatives—such as health-promoting university networks in Spain and Catalonia—have sought to foster healthier campus environments (8, 10, 11). The Catalan Network of Healthy Universities (Xarxa US.cat) (68), which includes the UOC, reflects this commitment. Nonetheless, numerous studies continue to report suboptimal dietary habits among university communities, including low adherence to the Mediterranean Diet (MD), insufficient fruit and vegetable intake, and high consumption of red and processed meat (19, 23, 24). These patterns frequently co-occur with insufficient PA, prolonged sedentary time, and stress-related behaviors, highlighting the need for integrated evaluations of lifestyle behaviors and diet in university settings (18, 19).

In addition to health, environmental sustainability has emerged as a key component of modern dietary recommendations, emphasizing dietary patterns that support both human health and environmental protection (7, 25). Universities increasingly incorporate sustainability principles into their health-promotion strategies, yet little is known about the extent to which their communities adhere to such dietary patterns. This highlights the importance of evaluating both healthy and sustainable eating behaviors in online university settings.

Several indices have been developed to evaluate adherence to healthy or sustainable dietary patterns, including the Mediterranean Diet Adherence Screener (MEDAS), the Healthy Eating Index (HEI), and the Planetary Health Diet Index (PHDI) (26–28). While MEDAS focuses on health-related adherence to the MD using simple frequency-based questions, HEI and PHDI require more detailed dietary data, including portion sizes, nutrient intakes, or linkage to external databases. Moreover, only PHDI explicitly integrates sustainability considerations. Their application in large online university populations remains limited.

Understanding lifestyle behaviors, including dietary patterns, is essential for designing targeted health promotion strategies and informing prioritisation of measures in online university monitoring initiatives. Therefore, this study aimed to examine health-related behavior patterns, including adherence to healthy and sustainable diets and its associations with PA, sedentary behavior, sleep, and emotional well-being, among students and staff at the UOC, a leading Spanish institution in online higher education.

Understanding lifestyle behaviors, including dietary patterns, is essential for designing targeted health promotion strategies and informing prioritisation of measures in online university monitoring initiatives. Specifically, this study addressed the following research questions: (1) What is the level of adherence to healthy and sustainable dietary patterns among students and staff of an online university? (2) How health-related behaviors—including physical activity, sedentary behavior, sleep quality, and emotional well-being differ between students and staff, and by gender? (3) How does the levels of dietary adherence differ across sociodemographic and health-related variables?

## Methods

### Study design and participants

**Figure 1:**
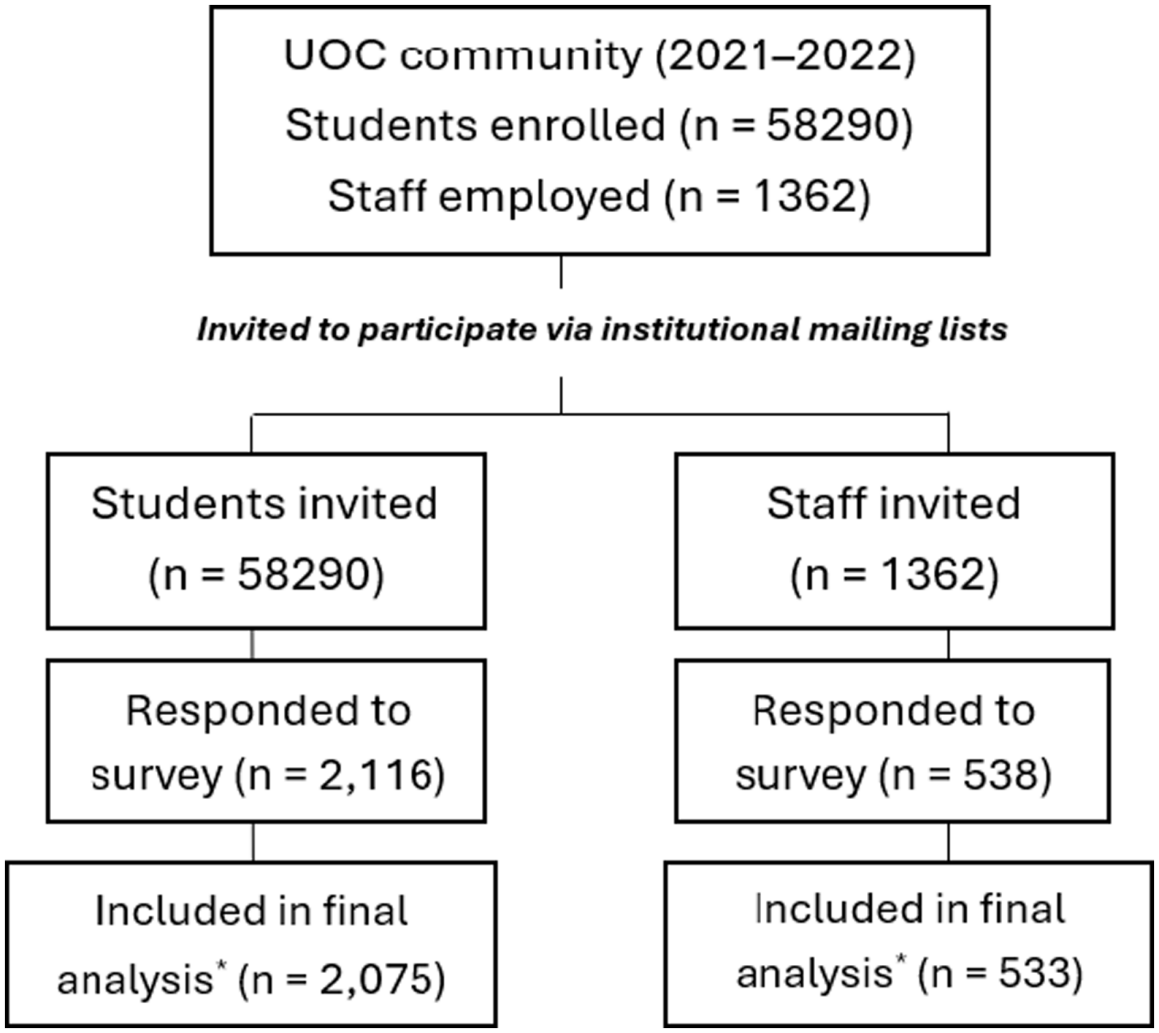
Flowchart of participant recruitment, exclusions, and final analytical sample. Minor exclusions due to incomplete questionnaires or implausible values.

This cross-sectional study was conducted during the 2021–2022 academic year within the UOC community, including students (undergraduate, master’s, postgraduate, and doctoral) and university staff (academic, research, administrative, and service personnel) (66). All members of the university community were invited to participate via institutional mailing lists and the virtual campus. The study protocol was approved by the Research Ethics Committee of UOC on 22 September 2021 (reference number: CE21-TE04). Participation was voluntary and anonymous for research purposes only, and all participants provided informed consent prior to completing the questionnaire. A participant flowchart describing the recruitment process, exclusions, and final analytical sample is presented in Figure 1.

### Data collection and measures

Data were collected through a structured, self-administered online survey (Qualtrics, Provo, UT, USA) (67) covering six domains: sociodemographic information, health status including body mass index (BMI), physical activity (PA) and sedentary behavior (SB), type of commuting, substance use (tobacco, alcohol and coffee), self-reported chronic conditions including migraine, hypertension and diabetes, and emotional well-being. Most items were drawn from validated instruments, including the International Physical Activity Questionnaire (29) for PA and SB (30), the General Health Questionnaire-12 (GHQ-12) for emotional well-being (31–33), and a semi-quantitative food frequency questionnaire (FFQ) in simplified and adapted from the Spanish population (70, 71) for dietary intake. Additional items were adapted from the Catalan Health Survey (ESCA) (34).

Several variables were recorded to improve group balance and interpretability. Sociodemographic variables included household composition, socioeconomic status, and, for staff, education level. Health status encompassed BMI categories and self-reported chronic conditions, which were grouped as follows: mental health (binary variable coded based on self-reported stress, anxiety, or depression), musculoskeletal disorders, migraines, and other chronic diseases; and functional limitations, based on six disability domains. Diet-related variables included self-reported adherence to plant-rich diets (PRD) (Mediterranean, vegetarian, vegan, flexitarian) or Western-style diets (WD) (frequent fast food and energy-dense nutrient-poor foods), as well as meal context (social meals, eating alone, or distracted eating) and frequency of shared meals. Caffeine intake was categorized according to weekly consumption. Lifestyle behaviors included physical activity (PA) levels, quantified using Metabolic Equivalent of Task (METs) values from the Compendium of Physical Activities (35) and categorized as low, moderate, or high; sedentary behavior, classified according to the Sedentary Behavior Research Network consensus (36) as active versus sedentary sitting time; occupational PA for staff; and sleep quality. Substance use covered alcohol (including binge drinking), tobacco, and other substances, with categories reflecting frequency of use. Mental well-being was assessed through GHQ-12, categorized into good, moderate, or poor. The GHQ-12 has been previously validated in the Spanish population, showing good internal consistency (Cronbach’s *α* > 0.80) and adequate construct validity in both general and working populations (37, 38). All detailed definitions and category thresholds are provided in Supplementary material.

The FFQ assessed the usual intake of 18 major food groups, with frequencies ranging from “more than 3 times/day” to “less than once/month”. This dietary intake data was used to assess adherence to the 2022 Spanish Agency for Food Safety and Nutrition (AESAN) guidelines (Healthy and Sustainable Dietary Recommendations). Based on the frequency of consumption, participants were classified into *inferior*, *optimal*, or *excessive* intake categories for each food group. In addition, participants were asked to self-assess how healthy they perceived their diet to be on a scale from 0 (not healthy at all) to 10 (very healthy).

### HEASUS scoring system

Existing diet quality indices—such as the Mediterranean Diet Adherence Screener (MEDAS) (28), the Healthy Eating Index (HEI) (27), or the Planetary Health Diet Index (PHDI) (26)—have greatly contributed to assessing diet quality in terms of health and, more recently, sustainability. These indices are often applied to FFQs but typically require detailed dietary information, such as daily gram intakes, servings, or nutrient densities. In contrast, the HEAlthy and SUStainable diet score (HEASUS) was developed based on the Spanish national food-based dietary guidelines (1), conceptually aligned with the EAT-Lancet framework (7), and designed to be applicable to large-scale online surveys that rely on broader frequency categories rather than detailed quantitative intake data. Its design provides construct validity by grounding the scoring directly in established national dietary recommendations, while maintaining a pragmatic and scalable structure suitable for large online populations in which detailed quantitative intake data are not available. This methodological approach is consistent with recent frameworks (39) that infer sustainability from the composition and balance of food groups rather than from product origin, acknowledging that food type contributes far more to environmental impact than transport or geographic source (40, 41).

HEASUS is a frequency-driven, gradient-based scoring system in which self-reported consumption frequencies of specific food groups are assigned proportional scores. The main food groups include fruits, vegetables, legumes, whole cereals, refined cereals, nuts, white meat, red meat, fish, eggs, and dairy. Each main group contributes a maximum score of 1, with individual components stratified to reflect substitution within the group. For example, cereals are divided into whole and refined types, which together sum 1, with whole grains contributing higher scores. Meat is divided into white and red meat contributing positively up to 1, with red meat scored positively only for low recommended consumption (≤1–2 times/week - ranging from 0 to 0.25), while white meat contributes progressively from 0 to 0.75 depending on consumption frequency, with higher scores for recommended intake (1–2 times/week) and moderate scores for occasional intake (1–3/month). This stratification allows HEASUS to capture not only absolute intake but also substitution patterns within food groups, particularly whole vs. refined cereals and white vs. red meat. The scoring ensures that recommended foods contribute more strongly to the index, while limiting the impact of low recommended consumption (e.g., red meat) on the total score.

Foods and beverages associated with adverse health outcomes or a high environmental impact — such as sugar-sweetened drinks, energy drinks, “light” soft drinks, pastries, fast food, salty snacks, and processed meats — contribute to a penalty score of approximately −1. Individual items are weighted proportionally to their relative impact. Processed meat is assigned a higher absolute weight (−0.4 to +0.4) due to its well-established negative effects (42, 43), while other items are assigned smaller weights (−0.1 to +0.1). Scores increase with lower consumption, reflecting minimal impact at occasional intake (e.g., ≤1/month) and progressively penalizing frequent consumption (e.g., ≥3/day). Positive scores reflect adherence to recommended consumption frequencies for beneficial foods, while negative scores indicate deviation from recommended patterns in detrimental foods. The total HEASUS score is the sum of all main food groups (maximum 9) plus the unhealthy and unsustainable foods and beverages component (maximum ±1), resulting in a theoretical range of −1 to +10, with higher values indicating healthier and more sustainable diets. Table 1 provides the complete scoring system for all food groups, including consumption frequency categories, corresponding scores, and the application of negative points for high intake of nutrient-poor foods, ensuring full reproducibility of the HEASUS index.

**Table 1:** Scoring criteria for the HEAlthy and SUStainable (HEASUS) index according to the main food groups frequency.

Food group	> 3 day	2 day	1 day	3–6 week	2 week	1 week	1–3 month	< 1 month	Score range
Main									
Refined cereals	0.17	0.25	0.17	0.17	0.11	0.11	0.07	0	0–0.25
Whole cereals	0.75	0.5	0.5	0.33	0.33	0.22	0.22	0	0–0.75
Legumes	1	1	1	1	0.67	0.44	0.3	0	0–1
White meat	0	0	0.15	0.33	0.75	0.75	0.75	0.72	0–0.75
Red meat	0	0	0	0.05	0.17	0.25	0.25	0.25	0–0.25
Fish	0	0.3	0.67	1	0.67	0.44	0.3	0	0–1
Eggs	0	0	0.67	1	0.67	0.44	0.3	0.3	0–1
Dairy	0	1	1	0.67	0.67	0.44	0.44	0.3	0–1
Salad/vegetables	1	0.67	0.44	0.3	0.2	0	0	0	0–1
Fruit	1	1	0.67	0.44	0.3	0.2	0	0	0–1
Nuts (plain)	0.3	0.44	0.67	1	0.67	0.44	0.3	0	0–1
High-dense nutrient-poor foods and beverages									
Sugar-sweetened drinks	–0.1	−0.08	−0.05	−0.04	−0.02	0.05	0.08	0.1	−0.1 − 0.1
Energy drinks	–0.1	−0.08	−0.05	−0.04	−0.02	0.05	0.08	0.1	−0.1 − 0.1
Light drinks	–0.1	−0.08	−0.05	−0.04	−0.02	0.05	0.08	0.1	−0.1 − 0.1
Salty snacks	–0.1	−0.08	−0.05	−0.04	−0.02	0.05	0.08	0.1	−0.1 − 0.1
Pastries	–0.1	−0.08	−0.05	−0.04	−0.02	0.05	0.08	0.1	−0.1 − 0.1
Fast food	–0.1	−0.08	−0.05	−0.04	−0.02	0.05	0.08	0.1	−0.1–0.1
Processed meat	−0.4	−0.20	−0.13	−0.09	−0.06	0.13	0.2	0.4	−0.4–0.4

Positive scores indicate desirable intake, negative scores indicate undesirable intake. Intermediate frequencies are assigned proportionally to create a gradient-based score. The total HEASUS score ranges theoretically from −1 to 10.

### Variables and data handling

Key sociodemographic and health-related variables were harmonized to ensure sufficient sample sizes across categories. Participants with extreme outliers in BMI or sleep duration (>3 × interquartile range (IQR)) were excluded. Detailed information on variable definitions, categorizations, and data handling procedures is provided in the Supplementary material. Missing data were minimal (<5%) and assumed to be missing at random; listwise deletion was applied.

### Statistical analysis

Descriptive analyses were conducted separately for students and staff. Categorical variables were compared using Chi-square or Fisher’s exact tests (when expected counts <5), while continuous variables were compared using Kruskal–Wallis tests followed by Dunn’s post-hoc tests with Bonferroni correction. Effect sizes were calculated using eta squared (η^2^) and interpreted as small (<0.06), moderate (0.06 − 0.14), or large (>0.14). Participants were categorized into tertiles of HEASUS adherence (low, medium, high). Sex was included as a covariate in all models to account for gender-specific differences in lifestyle and dietary behaviors.

To identify determinants of HEASUS adherence, multiple modeling strategies were evaluated, including linear, generalized linear, generalized additive, and quantile regression (QR) (44). Due to the non-normal distribution of HEASUS scores and the presence of outliers, QR was selected as the primary method. Models were estimated at the 25th, 55th, and 75th percentiles, allowing exploration of associations across the distribution of dietary adherence.

Covariates included in both student and staff models were selected based on prior descriptive and exploratory analyses (Spearman correlations, Kruskal–Wallis tests, and tertile-based comparisons) and included age, gender (excluding “Other”), BMI, self-reported adherence to dietary patterns (PRD and WD), PA, SB, emotional well-being, sleep quality, smoking, alcohol intake, coffee intake, main mode of transport, and type of study program for students or employment role for staff. PA was derived from self-reported weekly MET-minutes using the IPAQ scoring protocol, and categorized into low, moderate, and high PA. Student models additionally included meal context (e.g., eating alone or socially), GHQ-12 emotional well-being score, presence of migraine or chronic conditions, satisfaction with interpersonal relationships, type of student (e.g., master, degree), and recent substance use (binary indicator of any use of selected substances in the past month, separate from smoking or caffeine intake); staff models included education level (based on highest qualification attained), self-perceived health, and occupational PA (daily activity level related to work, distinguished from total PA). Variables included in descriptive reporting such as chronic conditions, migraine, and satisfaction with relationships were also documented in Supplementary materials. Bootstrap resampling (*n* = 1,000) was used to estimate robust standard errors and 95% confidence intervals. Analyses were conducted in R (v4.3.3), with significance set at *p* < 0.05. Participants were recruited via institutional mailing lists, with all invited individuals given the opportunity to participate.

## Results

### Participant characteristics

A total of 2,608 participants completed the online survey, including 2,075 students (mean age 35 ± 11 years) and 533 staff (mean age 45 ± 9 years), corresponding to response rates of 64% among students and 57% among staff. These participation rates are typical for online surveys in large academic populations (45) and may have been influenced by survey fatigue during the COVID-19 pandemic. Female participants predominated in both staff and students (70%), and all analyses were stratified by gender. Sociodemographic characteristics and health-related behaviors are summarized in Supplementary Table 1, with further details stratified by physical activity (PA) level in Supplementary Table 2.

### Dietary patterns and HEASUS scores adherence among students and staff

**Figure 2:**
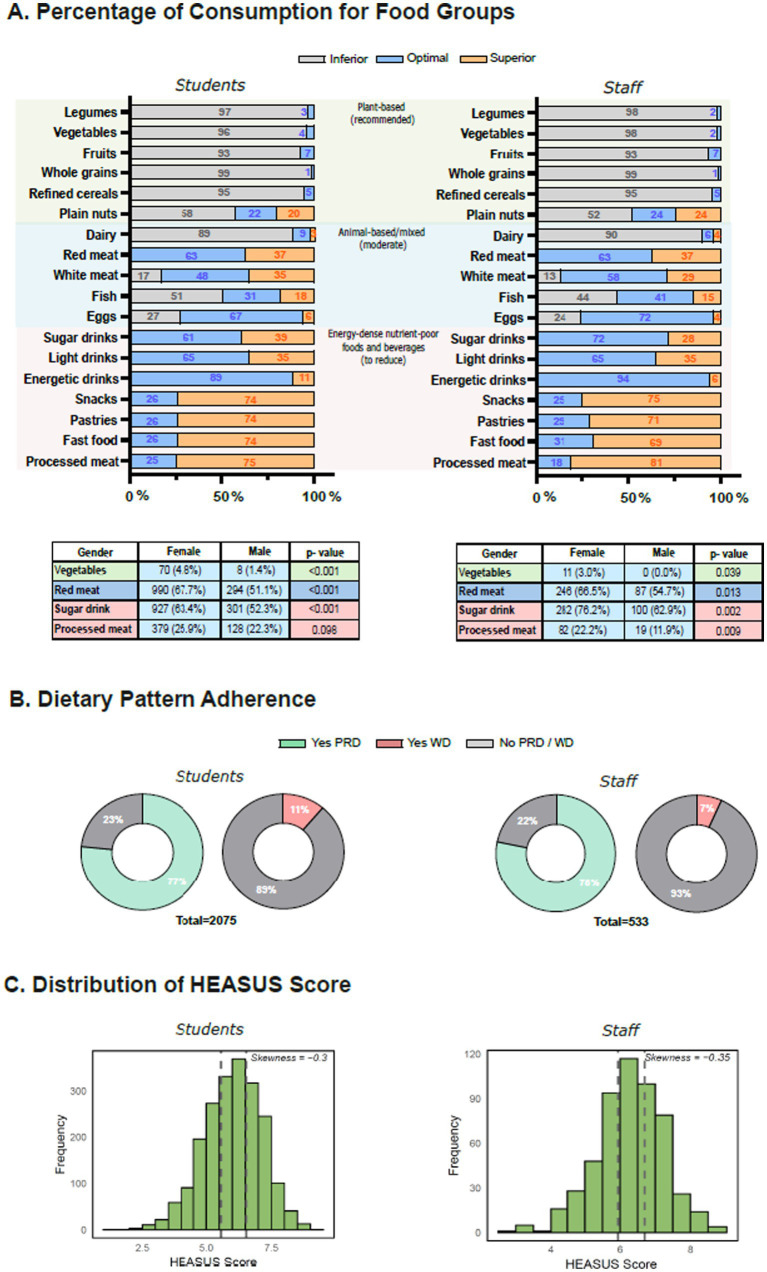
Dietary patterns, food group consumption, and HEASUS score distribution among students and staff. **(A)** Percentage of participants with optimal, inferior, or excessive intake for key food groups, based on national guidelines. Optimal food groups consumption by gender represented by *n* (%) of vegetables, read meat, sugar drinks and processed meat. Fisher’s exact test was used for the category Vegetables Chi-square (χ2). **(B)** Proportion of participants self-reporting adherence to plant-rich diet (PRD), western diet (WD), or neither. **(C)** Distribution of HEASUS scores, shown by frequencies, with skewness and subgroup comparison by tertiles for student and staff.

Overall adherence to healthy and sustainable dietary patterns, as assessed by the HEASUS index, was examined among students and staff, including differences by population group and gender. As shown in Figure 2A, most participants exceeded the recommended intake of processed meats, pastries, fast food, and snacks, while legumes, vegetables, whole grains, fruits, and plain nuts did not meet optimal intake in either population. Overall, there were no significant gender differences, except for vegetables, red meat, sugary drinks, and processed meat, where women were more likely than men to meet recommendations (vegetables: students *p* < 0.001, staff *p* = 0.039; red meat: students *p* < 0.001, staff *p* = 0.013; sugary drinks: students *p* < 0.001, staff *p* = 0.002; processed meat: students *p* = 0.098, staff *p* = 0.009).

Self-perceived healthy eating habits did not differ significantly by gender (students: women 7.0 ± 1.8 vs. men 6.9 ± 1.6, *p* = 0.061; staff: women 7.3 ± 1.7 vs. men 6.9 ± 1.6, p = 0.061; Supplementary Tables 1A,B). Self-reported adherence to a plant-rich diet (PRD) was higher among women compared to men (students: 79% vs. 71%, p = 0.002; staff: 81% vs. 71%, *p* = 0.02), whereas adherence to a Western-style diet (WD) was more common among men (students: *p* = 0.031; staff: *p* = 0.44). Figure 2B shows the proportion of participants adhering to PRD and WD in both populations.

Mean HEASUS scores indicated moderate adherence to healthy and sustainable dietary patterns, with females scoring higher than males (students: 6.1 vs. 5.8, *p* < 0.001; staff: 6.4 vs. 6.0, *p* < 0.01). Participants were categorized into tertiles of HEASUS adherence (T1: low, T2: medium, T3: high), and corresponding sociodemographic and health-related characteristics are presented in Table 2 (Full characteristics, including detailed lifestyle and health indicators, are reported in Supplementary Table 3). The distribution of HEASUS scores across populations is further illustrated in Figure 2C. Higher HEASUS adherence was associated with older age, female sex, and higher educational attainment (*p* < 0.05 for all). Participants in the highest tertile (T3) reported better self-perceived health, healthier eating habits, and lower prevalence of chronic conditions such as migraine (*p* < 0.001 in students; *p* = 0.647 in staff).

**Table 2:** Distribution of main variables across HEASUS tertiles adherence, separately calculated for each population group.

A. Students	T1 (*N* = 696)	T2 (*N* = 695)	T3 (*N* = 684)	[ALL] (*N* = 2075)	*p* value overall
HEASUS index range	≤ 5.56	5.57–6.54	≥ 6.55		
HEASUS score (range −1 to 10)	4.8 (± 0.66)	6.1 (± 0.28)	7.2 (± 0.50)	6.0 (± 1.1)	–
Gender					0.014
Male	217 (31%)	200 (29%)	158 (23%)	575 (28%)	
Female	469 (67%)	483 (69%)	511 (75%)	1,463 (71%)	
Others	10 (1%)	12 (2%)	15 (2%)	37 (2%)	
Age	33 (± 9.7)	36 (± 11)	37 (± 11)	35 (± 11)	< 0.001
Body mass index (BMI)	24 (± 4.7)	24 (± 4.3)	23 (± 3.7)	24 (± 4.3)	0.099
Student type					< 0.001
Undergraduate	476 (68%)	458 (66%)	395 (58%)	1,329 (64%)	
PhD	20 (3%)	17 (2%)	23 (3%)	60 (3%)	
Master/postgraduate	200 (29%)	220 (32%)	266 (39%)	686 (33%)	
Type of study					< 0.001
Arts/humanities	142 (20%)	131 (19%)	148 (22%)	421 (20%)	
Engineering	137 (20%)	112 (16%)	104 (15%)	353 (17%)	
Health	53 (8%)	58 (8%)	108 (16%)	219 (11%)	
Social/legal	364 (52%)	394 (57%)	324 (47%)	1,082 (52%)	
Self-perceived health					< 0.001
Regular or bad	138 (20%)	89 (13%)	75 (11%)	302 (15%)	
Good	311 (45%)	279 (40%)	250 (37%)	840 (40%)	
Very good	247 (35%)	327 (47%)	359 (52%)	933 (45%)	
Sleep quality					0.017
Regular or bad	332 (48%)	302 (43%)	274 (40%)	908 (44%)	
Good	225 (32%)	222 (32%)	226 (33%)	673 (32%)	
Very good	139 (20%)	171 (25%)	184 (27%)	494 (24%)	
Chronic mental health					< 0.001
No	275 (40%)	348 (50%)	344 (50%)	967 (47%)	
Yes	421 (60%)	347 (50%)	340 (50%)	1,108 (53%)	
Migraine (chronic)					< 0.001
No	480 (69%)	529 (76%)	534 (78%)	1,543 (74%)	
Yes	216 (31%)	166 (24%)	150 (22%)	532 (26%)	
Plant rich diet (PRD)					< 0.001
No	280 (40%)	134 (19%)	71 (10%)	485 (23%)	
Yes	416 (60%)	561 (81%)	613 (90%)	1,590 (77%)	
Western diet (WD)					< 0.001
No	570 (82%)	621 (89%)	651 (95%)	1842 (89%)	
Yes	126 (18%)	74 (11%)	33 (5%)	233 (11%)	
Self-perceived healthy eating habits (range 0 to 10)	5.8 (± 2.1)	7.1 (± 1.7)	7.9 (± 1.6)	6.9 (± 2.0)	< 0.001
Physical activity (PA) level					< 0.001
Low	236 (34%)	155 (22%)	105 (15%)	496 (24%)	
Moderate	361 (52%)	419 (60%)	410 (60%)	1,190 (57%)	
High	99 (14%)	121 (17%)	169 (25%)	389 (19%)	
Sitting hours per day	7.8 (± 3.4)	7.4 (± 3.2)	7.0 (± 3.3)	7.4 (± 3.3)	< 0.001
Sedentary behavior (SB)					< 0.001
Active	398 (57%)	444 (64%)	463 (68%)	1,305 (63%)	
Sedentary	298 (43%)	251 (36%)	221 (32%)	770 (37%)	
Alcohol consumption					0.022
Never	174 (25%)	140 (20%)	174 (25%)	488 (24%)	
Almost never	203 (29%)	182 (26%)	167 (24%)	552 (27%)	
Occasionally	198 (28%)	227 (33%)	227 (33%)	652 (31%)	
Weekly	121 (17%)	146 (21%)	116 (17%)	383 (18%)	
Smoking status					< 0.001
Non-smoker	521 (75%)	542 (78%)	575 (84%)	1,638 (79%)	
Occasional smoker	66 (9%)	64 (9%)	62 (9%)	192 (9%)	
Regular smoker	109 (16%)	89 (13%)	47 (7%)	245 (12%)	
Emotional well-being (GHQ-12)					0.004
Good	77 (11%)	99 (14%)	110 (16%)	286 (14%)	
Moderate	307 (44%)	331 (48%)	328 (48%)	966 (47%)	
Poor	312 (45%)	265 (38%)	246 (36%)	823 (40%)	
Tertiles were computed separately based on the HEASUS score distribution within the students population group. Values are presented as mean (SD) for continuous variables and *n* (%) for categorical variables. *p* value overall corresponds to global *p*-values obtained from comparison tests across students tertiles. Chronic mental health condition: binary indicator for having any of the mental conditions (stress, anxiety, depression); Migraine (chronic): binary indicator of self-reported diagnosis of migraine (yes/no) categorized as part of chronic conditions; Test: Kruskal–Wallis (K-W) test used for continuous variables and Chi-square (χ2) for categorical variables. Complete data for all variables are provided in Supplementary Table 1.					

B. Staff	T1 (*N* = 182)	T2 (*N* = 174)	T3 (*N* = 177)	[ALL] (*N* = 533)	*p* value overall
HEASUS index range	≤ 5.93	5.94–6.70	≥ 6.71	–	–
HEASUS score (range −1 to 10)	5.3 (± 0.61)	6.3 (± 0.21)	7.3 (± 0.46)	6.3 (± 0.95)	–
Gender					< 0.001
Male	74 (41%)	52 (30%)	33 (19%)	159 (30%)	
Female	105 (58%)	122 (70%)	143 (81%)	370 (69%)	
Others	3 (2%)	0 (0%)	1 (1%)	4 (1%)	
Age	44 (± 8.4)	45 (± 9.4)	45 (± 9.7)	45 (± 9.2)	0.545
Body mass index (BMI)	24 (± 3.9)	24 (± 3.6)	23 (± 3.6)	24 (± 3.7)	0.461
Work type					< 0.001
PAS	127 (70%)	104 (60%)	74 (42%)	305 (57%)	
PDI	28 (15%)	40 (23%)	48 (27%)	116 (22%)	
PDC	27 (15%)	30 (17%)	55 (31%)	112 (21%)	
Self-perceived health					< 0.001
Regular	22 (12%)	9 (5%)	8 (5%)	39 (7%)	
Good	91 (50%)	89 (51%)	64 (36%)	244 (46%)	
Very good	69 (38%)	76 (44%)	105 (59%)	250 (47%)	
Sleep quality					0.341
Regular or bad	93 (51%)	91 (52%)	86 (49%)	270 (51%)	
Good	13 (7%)	16 (9%)	24 (14%)	53 (10%)	
Very good	76 (42%)	67 (39%)	67 (38%)	210 (39%)	
Chronic mental health					0.358
No	159 (87%)	152 (87%)	162 (92%)	473 (89%)	
Yes	23 (13%)	22 (13%)	15 (8%)	60 (11%)	
Migraine (chronic)					0.647
No	165 (91%)	161 (93%)	165 (93%)	491 (92%)	
Yes	17 (9%)	13 (7%)	12 (7%)	42 (8%)	
Plant rich diet (PRD)					< 0.001
No	63 (35%)	40 (23%)	13 (7%)	116 (22%)	
Yes	119 (65%)	134 (77%)	164 (93%)	417 (78%)	
Western diet (WD)					< 0.001
No	156 (86%)	168 (97%)	173 (98%)	497 (93%)	
Yes	26 (14%)	6 (3%)	4 (2%)	36 (7%)	
Self-perceived healthy eating habits (range 0 to 10)	6.4 (± 1.8)	7.4 (± 1.4)	7.8 (± 1.6)	7.2 (± 1.7)	< 0.001
Physical activity (PA) level					0.105
Low	36 (20%)	24 (14%)	17 (10%)	77 (14%)	
Moderate	123 (68%)	127 (73%)	136 (77%)	386 (72%)	
High	23 (13%)	23 (13%)	24 (14%)	70 (13%)	
Sitting hours per day	8.3 (± 2.8)	8.1 (± 2.9)	7.9 (± 2.7)	8.1 (± 2.8)	0.044
Sedentary behavior (SB)					0.216
Active	89 (49%)	82 (47%)	99 (56%)	270 (51%)	
Sedentary	93 (51%)	92 (53%)	78 (44%)	263 (49%)	
Alcohol consumption					0.205
Never	19 (10%)	28 (16%)	29 (16%)	76 (14%)	
Almost never	28 (15%)	33 (19%)	31 (18%)	92 (17%)	
Occasionally	68 (37%)	70 (40%)	68 (38%)	206 (39%)	
Weekly	67 (37%)	43 (25%)	49 (28%)	159 (30%)	
Smoking status					0.011
Non-smoker	129 (71%)	143 (82%)	150 (85%)	422 (79%)	
Occasional smoker	23 (13%)	13 (7%)	15 (8%)	51 (10%)	
Regular smoker	30 (16%)	18 (10%)	12 (7%)	60 (11%)	
Emotional well-being (GHQ-12)					0.8
Good	15 (8%)	18 (10%)	18 (10%)	51 (10%)	
Moderate	116 (64%)	107 (61%)	117 (66%)	340 (64%)	
Poor	51 (28%)	49 (28%)	42 (24%)	142 (27%)	
Tertiles were computed separately based on the HEASUS score distribution within the staff population group. Values are presented as mean (SD) for continuous variables and *n* (%) for categorical variables. *p* value overall corresponds to global *p*-values obtained from comparison tests across staff tertiles. Work type: PAS (Administrative and Service Staff), PDI (Teaching and Research Staff), and PDC (Administrative and Service Staff); Chronic mental health condition: binary indicator for having any of the mental conditions (stress, anxiety, depression); Migraine (chronic): binary indicator of self-reported diagnosis of migraine (yes/no) categorized as part of chronic conditions. Test: Kruskal–Wallis (K-W) test used for continuous variables and Chi-square (χ2) for categorical variables. Complete data for all variables are provided in Supplementary Table 1.					

T1: Low adherence; T2: Medium adherence; T3: High adherence.

Participants with higher HEASUS scores were also more likely to follow a PRD (from 60% in T1 to 90% in T3, *p* < 0.001) and less likely to follow a WD (from 18% in T1 to 5% in T3, *p* < 0.001). This gradient supports the internal validity of the HEASUS index as an indicator of overall diet quality and sustainability.

### Differences in lifestyle behaviors by HEASUS adherence

This section explores the association between levels of HEASUS adherence and health-related behaviors, including physical activity, sedentary behavior, sleep quality, emotional well-being, and substance use, stratified by population group and gender. The majority of both populations complied with PA recommendations. Males were more likely to engage in high levels of PA: 23% in students (*p* < 0.002) and 17% in staff (*p* < 0.08), whereas females showed a more balanced distribution across “Low” and “Moderate” activity levels. Higher levels of PA were consistently associated with higher HEASUS scores and healthier lifestyle behaviors such as better quality of sleep and emotional well-being (Table 2; Supplementary Table 2). Among students, HEASUS scores increased progressively across PA levels (5.6 ± 1.1, 6.1 ± 1.1, and 6.3 ± 1.1 for low, moderate, and high PA, *p* < 0.001), accompanied by a gradual reduction in BMI (24.6 ± 5.0, 23.7 ± 4.1, and 23.2 ± 3.6, *p* < 0.001). Among staff, HEASUS scores and self-perceived healthy eating habits also increased with PA, while BMI did not differ significantly across PA levels (*p* = 0.83). Supplementary Figure 1 illustrates these relationships, showing decreasing BMI and increasing Metabolic Equivalent of Task (METs) values across HEASUS tertiles. Participants reporting higher PA also perceived their diet as healthier (students *p* < 0.001; staff *p* = 0.004) and their general health (students and staff *p* < 0.001) and sleep quality as better (students *p* < 0.017; staff *p* = 0.341). Supplementary Figure 2 presents correlation patterns among numerical variables, including age, General Health Questionnaire-12 (GHQ-12) scores, self-reported healthy eating habits, hours of sleep, and sitting time per day.

Average sitting time was slightly higher in male students (7.6 ± 3.3 h) than females (7.3 ± 3.2 h, *p* = 0.06), with no significant gender differences among staff (Supplementary Table 1). Sitting time decreased progressively across HEASUS tertiles in both populations, with students reporting 7.8 ± 3.4, 7.4 ± 3.2, and 7.0 ± 3.3 h/day in T1, T2, and T3, respectively (*p* < 0.001), and staff showing a similar but smaller trend (8.3 ± 2.8, 8.1 ± 2.9, 7.9 ± 2.7, *p* = 0.044). Active commuting (walking or cycling) was more frequent among highly active individuals and increased across HEASUS tertiles in students (T1: 20%, T2: 22%, T3: 26%; *p* = 0.021) and staff (T1: 32%, T2: 35%, T3: 40%; *p* < 0.001). Conversely, sedentary commuting (car or motorbike) was more common among those with lower HEASUS scores.

Poor emotional well-being (GHQ-12) and poor sleep quality was more prevalent among female students (43% vs. 30.8% males, *p* < 0.001 for poor GHG12 score and 46% vs. 37% males, *p* < 0.001 for poor sleep quality), whereas in staff it did not differ. Weekly consumption of alcohol was more prevalent in males of both populations (27% vs. 14%) females students (*p* < 0.001) and 41% vs. 25% in female staff (*p* = 0.001) (Supplementary Table 1).

Higher HEASUS scores were associated with better emotional well-being, healthier lifestyle behaviors, and lower prevalence of substance use, particularly among students. In students, the highest tertile (T3) showed significantly lower prevalence of poor emotional well-being (36% vs. 45% in T1, *p* = 0.004), fewer chronic mental health conditions (50% vs. 60%, *p* < 0.001), higher satisfaction with interpersonal relationships (76% vs. 63%, *p* < 0.001), and higher self-perceived healthy eating habits (7.9 vs. 5.8, *p* < 0.001) compared with the lowest tertile. Non-smoking status increased across tertiles (T1: 75% vs. T3: 84%, *p* < 0.001), while occasional or weekly alcohol consumption and past-month substance use decreased (substance use: 9% vs. 5%). Students in higher tertiles also reported better sleep quality and slightly higher coffee consumption. Among staff, higher HEASUS tertiles were significantly associated with greater self-perceived healthy eating habits (6.7 vs. 7.6, *p* < 0.001) and lower regular smoking prevalence (16% vs. 7%, *p* = 0.011), whereas emotional well-being, alcohol and substance use, and satisfaction with relationships showed minimal variation (all *p* > 0.05).

### Differences in dietary adherence levels across sociodemographic and health-related factors

To examine how dietary adherence levels differ across sociodemographic and health-related variables, quantile regression analyses were conducted. These analysis confirmed and extended the patterns observed across HEASUS tertiles (Table 2), identifying consistent behavioral and demographic determinants of HEASUS adherence. Positive predictors of higher HEASUS scores included self-reported adherence to a PRD (strongest predictor), higher PA levels (particularly among students), enrollment in health-related study programs (students), and active commuting (staff, lower HEASUS quantiles). In contrast, negative predictors included self-reported adherence to a WD, male gender, overweight status, smoking, passive commuting, and low-to-moderate coffee intake. Notably, sleep quality, sedentary time, and meal conviviality (i.e., degree of socialization during meals) were not consistently associated with HEASUS. Analyses were performed using quantile regression at the 25th, 55th, and 75th percentiles of HEASUS scores to capture associations across the distribution and account for non-normality and outliers (see Supplementary Table 4).

## Discussion

### Dietary patterns and HEASUS adherence in an online university setting

This study provides a broad characterization of health-related and sustainability behaviors in a large online university population, offering insights into lifestyle challenges and opportunities in digital higher education contexts. Overall, dietary pattern reflected moderate adherence to national dietary recommendations (1), with insufficient intake of plant-rich foods—particularly vegetables, fruits, legumes, and nuts—and excessive consumption of red and processed meats, pastries, and energy-dense nutrient-poor foods and beverages. These patterns not only compromise nutritional quality but also contribute to higher environmental footprints, highlighting the dual challenge and opportunity of improving health and planetary health in university populations.

The HEASUS index was developed based on the AESAN Food-Based Dietary Guidelines (FBDGs) (1), and in turn inspired by the EAT-Lancet recommendations for healthy and sustainable diets (7). Its frequency-driven design allows for a culturally adapted, pragmatic assessment suitable for large online populations where detailed quantitative intake data may not be available.

Quantile regression analyses indicated that lifestyle determinants had a stronger influence on HEASUS adherence among individuals with lower levels of dietary adherence. In particular, physical activity, sedentary behavior, and sleep quality showed the strongest associations with HEASUS scores in the lowest adherence quartile (*τ* = 0.25). This pattern suggests that individuals with poorer dietary adherence may benefit most from targeted lifestyle interventions. Overall, higher HEASUS scores were consistently associated with healthier dietary patterns, greater physical activity, better sleep quality, improved emotional well-being, and a lower prevalence of risk behaviors such as smoking and frequent alcohol consumption. Together, these findings help identify priority groups for health promotion and sustainability-focused interventions.

### Factors health-related behaviors associated with HEASUS adherence

Higher HEASUS adherence was consistently associated with healthier lifestyle behaviors, including greater physical activity, better sleep quality, improved emotional well-being, and a lower prevalence of risk behaviors. Within this online university population, both lifestyle behaviors and sociodemographic characteristics showed substantial variability, highlighting the heterogeneous nature of dietary adherence and its associated determinants. Overall, students and staff reported moderate levels of PA, mixed commuting habits, diverse sleep quality, and generally low prevalence of substance use such as smoking or frequent alcohol consumption. These patterns are consistent with previous research linking dietary behaviors, PA, sleep, and emotional well-being among university students and staff (21, 46–48).

When stratified by HEASUS adherence, the general patterns were accentuated and differences in gender were evident. Women generally reported higher diet quality (higher HEASUS scores) but also more stress and greater sedentary behavior, consistent with previous findings on gender differences in diet and physical activity (46, 49). Men displayed higher prevalence of overweight, poorer diet quality, and more frequent substance use, including weekly alcohol use and binge drinking. These findings align with prior evidence on gendered differences in health behaviors and highlight the need for gender-sensitive interventions addressing both dietary behaviors and lifestyle stressors in university settings (50, 51, 66). Poor sleep quality and stress were also more frequent among women and those with lower HEASUS scores, supporting previous research on the bidirectional relationship between emotional well-being, sleep, and diet quality (52, 53). Additionally, perceived stress has been shown to influence health behaviors, including diet and PA (54).

Although overall HEASUS adherence was moderate in both groups, staff consistently showed slightly higher scores than students, which may reflect differences in age, work routines, and health awareness. Enrollment in health-related degrees for students, older age, and active commuting were positive predictors of higher HEASUS adherence, while self-reported WD pattern, overweight status, and passive commuting were associated with lower adherence, reinforcing previous literature linking lifestyle, occupation, and knowledge with dietary quality (55–57). Among staff, these trends were less pronounced, though a similar pattern of more active commuting and lower sedentary behavior was observed in higher HEASUS tertiles.

### Implications

Our findings indicate that targeting individuals with the lowest HEASUS adherence can maximize the effectiveness of health promotion programs, generating cascading benefits across multiple health domains, including physical activity, diet, sleep, and emotional well-being. For example, promoting PA not only improves fitness and metabolic health (58) but is also associated with healthier dietary choices, better sleep, and enhanced emotional well-being. Online and hybrid university settings offer a unique opportunity to implement scalable interventions—such as digital nudges, gamification, plant-based challenges, and real-time dietary feedback—which have been shown to influence health behaviors, including diet, PA, and SB (59, 60). Given the observed gender differences in stress, sleep, and SB, gender-responsive strategies are essential. Promoting plant-rich diets (PRD) further supports individual health while contributing to environmental sustainability, linking dietary interventions to broader planetary health goals (5, 7).

Additionally, the observed dietary patterns have implications beyond individual health. Higher HEASUS adherence, characterized by greater intake of PRD and lower consumption of processed meats and energy-dense nutrient-poor foods, supports principles of planetary health diets, highlighting the dual benefit of diet modification for both health and environmental sustainability. Comparable indices, such as the Sustainable Healthy Diet Index (SHDI), have similarly integrated health and sustainability considerations in European and North African populations (39). Unlike SHDI, which relies on gram-based conversion and detailed intake data, HEASUS is a pragmatic, frequency-based tool for a broader population enabling scalable assessment and intervention planning. Integrating dietary interventions with active commuting, sleep hygiene, and emotional well-being support could therefore simultaneously promote human and planetary health in university settings.

### Strengths and limitations

Key strengths of this study include the application of a culturally adapted, frequency-based dietary index constructed directly from AESAN FBDGs and conceptually aligned with the EAT-Lancet framework. It simultaneously evaluates lifestyle, health-related behaviors including emotional well-being, and sociodemographic factors in a large online university population.

Limitations include reliance on self-reported data, which may introduce recall or social desirability bias, and the cross-sectional design, which limits causal inference. The heterogeneity of the population—spanning diverse ages, academic programs, and work roles—adds complexity but also enhances generalizability to other online higher education settings.

Data collection took place one year after the COVID-19 pandemic lockdown in Spain, which may have influenced lifestyle behaviors and mental well-being and should be considered when interpreting the findings. However, many of the structural conditions observed—such as remote learning, flexible schedules, prolonged screen time, and reduced active commuting—remain intrinsic to online university settings. Therefore, the findings remain highly relevant for understanding lifestyle behaviors in digital higher education environments (61).

### Future directions

Future research should explore the implementation of targeted, scalable interventions that simultaneously address diet quality, PA, sedentary behavior, sleep, and emotional well-being. Interventions could leverage digital health platforms, gamification, and real-time feedback (62–64) to increase engagement, particularly among those in the lowest HEASUS tertile. Digital nudging interventions have been shown to increase short PA breaks among home-studying students (65). Longitudinal monitoring of lifestyle behaviors is needed to track changes over time and evaluate the potential causal effects of health promotion interventions.

While HEASUS was developed for online university populations in Spain, its pragmatic, frequency-based design enables adaptation to other online cohorts, including adult learners, or remote employees. Such adaptations could facilitate cross-population comparisons of adherence to healthy and sustainable diets. Longitudinal studies are needed to evaluate changes over time and the potential effects of health promotion interventions. Integrating environmental impact assessments, such as life cycle analyses or carbon footprints, would further strengthen the sustainability dimension of dietary interventions.

## Conclusion

Despite moderate adherence to a healthy and sustainable dietary pattern overall based on HEASUS, substantial gaps exist in levels of PA and emotional well-being. Findings highlight the need for integrated, gender-sensitive interventions that enhance PRD consumption, reduce energy-dense nutrient-poor foods, support emotional wellbeing and encourage active daily routines.

## Data Availability

The raw data supporting the conclusions of this article will be made available by the authors, without undue reservation.

## Glossary

AESAN: Agency for Food Safety and Nutrition
AIC: Akaike Information Criterion
BIC: Bayesian Information Criterion
BMI: Body Mass Index
DQI-I: Diet Quality Index–International
ESCA: US. Cat health commission
FFQ: Food Frequency Questionnaire
GAM: Generalized Additive Models
GDQS: Global Diet Quality Score
GHQ-12: General Health Questionnaire-12 item
GLM: Generalized Linear Models
HEASUS: HEAlthy and SUStainable Diet Index
HEI: Healthy Eating Index
IPAQ: Physical Activity Questionnaire
IQR: Interquartile range
LM: Linear Regression
MEDAS: Mediterranean Diet Adherence Screener
METs: Metabolic Equivalent of Task
PA: Physical Activity
PAS: Administrative and Service Staff
PDC: Administrative and Service Staff
PDI: Teaching and Research Staff
PHDI: Planetary Health Diet Index
PRD: Plant-Rich Diet
pseudo-R^2^: Pseudo Coefficient of Determination
QR: Quantile regression
SB: Sedentary Behavior
FBDG: Food Based Dietary Guidelines
UOC: Open University of Catalonia (UOC)
US. Cat: Catalan Network of Healthy Universities
WD: Western-style Diet
*β*: Regression coefficient
η^2^: Effect sizes (eta squared)
*ρ*: Correlation Coefficient
*τ*: Quantile
