# Supplementary material for: Adherence to healthy and sustainable diets and health-related behaviors in a Spanish online university setting
Source: Front Nutr. 2026 Mar 12;13:1757733. doi: 10.3389/fnut.2026.1757733 (PMC13017255; doi:10.3389/fnut.2026.1757733)
Supplement: Supplementary file 1 [file Table_1.docx]

***Supplementary Material***

# Supplementary Data

*Variable Coding and Data Handling*

Categorical variables with sparse categories were collapsed to improve group balance. Participants with extreme outliers in Body Mass Index (BMI) or total hours of sleep were excluded (values beyond three times the interquartile range, IQR). The following variables were categorize as followed: Household Composition: Participants were grouped as family/partner, shared accommodation, living alone, or other relatives/others; Socioeconomic Status: Self-perceived financial situation was assessed on a 5-point scale, categorized as worse (1–2), average (3), and better (4–5); Education (staff only): Categorized as low (secondary or less, including “other studies”), medium (vocational training or first university cycle), and high (second cycle/postgraduate); Chronic Conditions: Self-reported; dichotomized into yes/no, then grouped as mental health (stress, anxiety, depression), musculoskeletal disorders, migraines, or other chronic diseases (e.g., hypertension, diabetes, cardiovascular); Disability: Assessed in six domains (vision, hearing, mobility, physical function, basic daily activities, need for assistive devices). Responses were binary (no limitation vs. limitation), with “Don’t know” or “Prefer not to answer” treated as missing. A summary variable indicated the presence of any functional limitation. Plant-Rich Diets (PRD): Adherence to Mediterranean, vegetarian, vegan, or flexi-vegetarian diets over the past year. Western-Style Diets (WD): High consumption of fast food and energy-dense nutrient-poor foods (snacks, pastries). Meal Context: Categorized as social (eating with others while talking), alone (eating alone without distractions), and distracted (eating alone while reading, using screens, or working). Weekly shared meals were calculated and categorized as high (≥5/week), medium (2–4/week), and low (<2/week). Caffeine Intake: Coffee and energy drinks were categorized as low (≤1/week), moderate (2–6/week or once/day), or high (≥2/day). Physical Activity (PA): Assessed via IPAQ. Metabolic Equivalent of Task (METS) calculated as:

$$METS=8\times(Days of vigorous-intensity activity)\times(Minutes of vigorous-intensity activity)+4\times(Days of moderate-intensity activity)\times(Minutes of moderate-intensity activity)+3.3\times(Days of walking)\times(Walking minutes)$$

PA levels categorized as low (<600 METS-min/week), moderate (600–3,000), or high-vigorous (>3,000); Sedentary Behavior: Self-reported sitting time classified as active (0.5–8 h/day) or sedentary (>8 h/day); Occupational PA (staff): Single-item question on daily movement, grouped into low (mostly sitting/standing), moderate (frequent walking), or high (physically demanding); Mental Well-being: GHQ-12 scored 0–36, with good (0–11), moderate (12–19), and poor (20–36) mental health. Positive items were reverse-coded; Satisfaction with Interpersonal Relationships: Rated 1–5, categorized as low (dissatisfied), medium (acceptable), or high (satisfied); Alcohol Consumption: Quantity per occasion (1 drink, 2–3, ≥4) and binge drinking (≥6 units) categorized as never, occasional (monthly or less), or frequent (weekly or more); Smoking: Non-smoker, occasional (<weekly), regular (≥2/week); Substance Use: Categorized as never, lifetime, past year, past month. Students reported six substances, staff reported two; “Prefer not to answer” coded as missing. A binary variable for past-month use was also created.

**Statistical Analysis**

Categorical variables with expected cell counts <5 were analyzed using Fisher’s exact test. Effect sizes were calculated via Eta Squared (η²) from Kruskal–Wallis statistics, interpreted as: small (<0.06), moderate (0.06–0.14), or large (>0.14).

$$\eta^{2}=\frac{H}{N-1}$$

where 𝐻 is the Kruskal-Wallis test statistic and 𝑁 is the total sample size.

To identify predictors of HEASUS scores, several modeling approaches were compared (linear regression, generalized linear models, generalized additive models, and quantile regression). Model selection considered Akaike Information Criterion (AIC), Bayesian Information Criterion (BIC), Pseudo Coefficient of Determination (pseudo-R²), and residual diagnostics (not shown).

Due to non-normal distribution (Shapiro–Wilk p < 0.001) and presence of outliers, quantile regression (QR) was selected for robustness. QR models were fitted at the 25th, 55th, and 75th percentiles (τ=0.25,0.55,0.75\tau = 0.25, 0.55, 0.75τ=0.25,0.55,0.75):

$$Q\tau(HEASUS\mid X)=\beta0(\tau)+\beta1(\tau)X1+\beta2(\tau)X2+\cdots+\epsilon$$

where $Q\tau(HEASUS\mid X)$ is the vector of covariates (see Methods section) and $X$ denotes the conditional quantile of HEASUS at percentile $\in\{0.25,0.55,0.75\}$ Standard errors were estimated via bootstrapping (n = 1,000) using the *quantreg* package in R (v4.3.3). Full regression outputs and bivariate analyses are included in **Supplementary Tables 5–6**, and distributional patterns of HEASUS by tertiles are illustrated in **Supplementary Figure 1**.

# Supplementary Figures and Tables


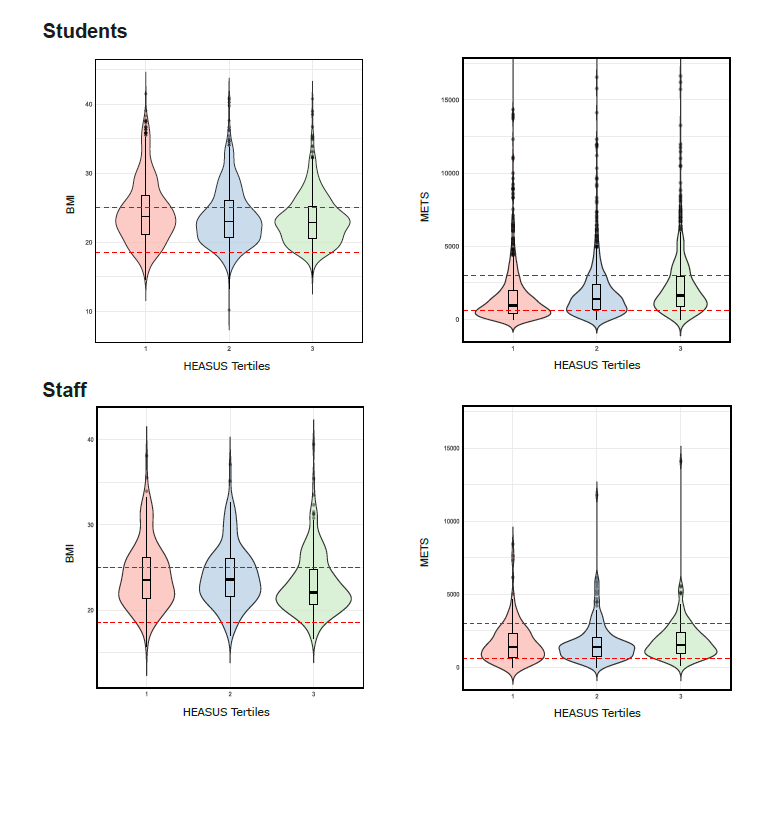
**Supplementary Figure 1.** **Distribution of HEASUS Score tertiles by Body Mass Index (BMI) and Metabolic Equivalent of Task (METs) among students (Top) and staff (Bottom).** Violin plots show the distribution of BMI and METs across HEASUS tertiles, with overlaid boxplots. Red dashed lines mark reference thresholds for BMI (18.5, 25) and METs (600, 3000). Extreme METs values (>17,000) excluded for clarity but included in analysis. All data are stratified by student/staff groups.


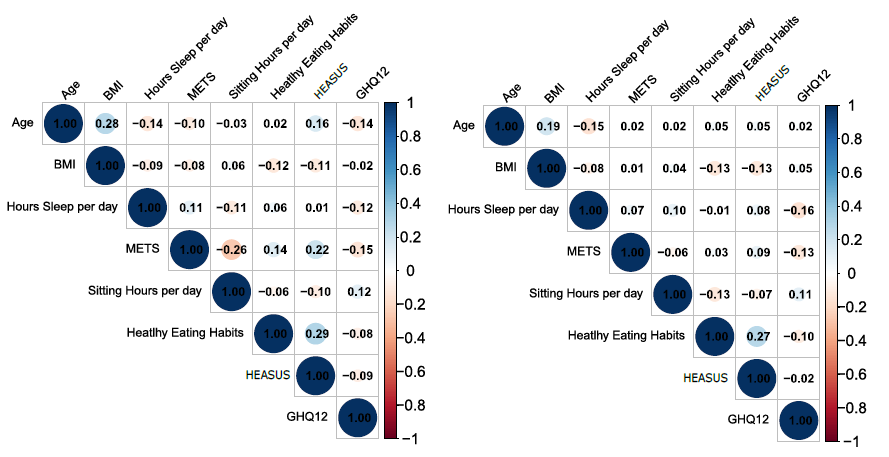


**Supplementary Figure 2:** Correlation matrix of numerical variables among students (left) and staff (right). Spearman’s rank correlation coefficients (ρ) are represented with a color gradient: blue = positive correlation, red = negative correlation. Darker colors indicate stronger correlations. BMI: Body Mass Index; METS: Metabolic Equivalent of Task; Healthy Eating Habits: self-reported healthy eating habits; HEASUS: HEAlthy and SUStainable diets; GHQ-12: General Health Questionnaire-12.

**Supplementary Table 1. Characteristics on health, lifestyle, and dietary patterns of the university community, by gender.**

1. **Students**

|  | **[ALL]**  **N=2075** | **Female**  **N=1463** | **Male**  **N=575** | **Others**  **N=37** | **p value overall** |
| --- | --- | --- | --- | --- | --- |
| **Age** | 35.4 (10.6) | 34.3 (10.1) | 38.3 (11.3) | 35.5 (11.9) | <0.001 |
| **Body Mass Index (BMI)** | 23.8 (4.3) | 23.3 (4.3) | 25.3 (3.8) | 23.4 (3.2) | <0.001 |
| **Student type:** |  |  |  |  | 0.19 |
| Undergraduate | 1329 (64.0%) | 941 (64.3%) | 360 (62.6%) | 28 (75.7%) |  |
| PhD | 60 (2.9%) | 36 (2.5%) | 23 (4.0%) | 1 (2.7%) |  |
| Master/postgraduate | 686 (33.1%) | 486 (33.2%) | 192 (33.4%) | 8 (21.6%) |  |
| **Type of study:** |  |  |  |  | . |
| Arts/Humanities | 421 (20.3%) | 308 (21.1%) | 100 (17.4%) | 13 (35.1%) |  |
| Engineering | 353 (17.0%) | 126 (8.6%) | 221 (38.4%) | 6 (16.2%) |  |
| Health | 219 (10.6%) | 168 (11.5%) | 48 (8.3%) | 3 (8.1%) |  |
| Social/Legal | 1082 (52.1%) | 861 (58.9%) | 206 (35.8%) | 15 (40.5%) |  |
| **Place of residence:** |  |  |  |  | <0.001 |
| Catalonia | 1246 (60.0%) | 921 (63.0%) | 302 (52.5%) | 23 (62.2%) |  |
| Spain or Other | 829 (40.0%) | 542 (37.0%) | 273 (47.5%) | 14 (37.8%) |  |
| **Household Composition:** |  |  |  |  | . |
| Family or Partner | 1685 (81.2%) | 1204 (82.3%) | 456 (79.3%) | 25 (67.6%) |  |
| Shared Accommodation | 89 (4.3%) | 61 (4.2%) | 24 (4.2%) | 4 (10.8%) |  |
| Alone | 227 (10.9%) | 141 (9.6%) | 82 (14.3%) | 4 (10.8%) |  |
| Other Relatives and Others | 74 (3.6%) | 57 (3.9%) | 13 (2.3%) | 4 (10.8%) |  |
| **Work status:** |  |  |  |  | <0.001 |
| Unemployed | 399 (19.2%) | 288 (19.7%) | 102 (17.7%) | 9 (24.3%) |  |
| Part Time | 377 (18.2%) | 300 (20.5%) | 66 (11.5%) | 11 (29.7%) |  |
| Full Time | 1299 (62.6%) | 875 (59.8%) | 407 (70.8%) | 17 (45.9%) |  |
| **Self-perceived health:** |  |  |  |  | 0.002 |
| Regular or Bad | 302 (14.6%) | 220 (15.0%) | 72 (12.5%) | 10 (27.0%) |  |
| Good | 840 (40.5%) | 613 (41.9%) | 210 (36.5%) | 17 (45.9%) |  |
| Very Good | 933 (45.0%) | 630 (43.1%) | 293 (51.0%) | 10 (27.0%) |  |
| **Sleeping hours per day** | 7.1 (1.0) | 7.1 (1.0) | 7.0 (1.0) | 7.2 (1.4) | 0.332 |
| **Sleep quality:** |  |  |  |  | <0.001 |
| Regular or Bad | 908 (43.8%) | 678 (46.3%) | 215 (37.4%) | 15 (40.5%) |  |
| Good | 673 (32.4%) | 465 (31.8%) | 191 (33.2%) | 17 (45.9%) |  |
| Very Good | 494 (23.8%) | 320 (21.9%) | 169 (29.4%) | 5 (13.5%) |  |
| **Mental health condition:** |  |  |  |  | <0.001 |
| No | 967 (46.6%) | 609 (41.6%) | 345 (60.0%) | 13 (35.1%) |  |
| Yes | 1108 (53.4%) | 854 (58.4%) | 230 (40.0%) | 24 (64.9%) |  |
| **Migraine (chronic):** |  |  |  |  | <0.001 |
| No | 1543 (74.4%) | 1013 (69.2%) | 503 (87.5%) | 27 (73.0%) |  |
| Yes | 532 (25.6%) | 450 (30.8%) | 72 (12.5%) | 10 (27.0%) |  |
| **Any disability:** |  |  |  |  | 0.518 |
| No | 1870 (92.1%) | 1318 (92.3%) | 522 (91.7%) | 30 (88.2%) |  |
| Yes | 161 (7.9%) | 110 (7.7%) | 47 (8.3%) | 4 (11.8%) |  |
| **HEASUS index (range -1 to 10)** | 6.0 (1.1) | 6.1 (1.1) | 5.8 (1.1) | 6.2 (0.9) | < 0.001 |
| **Plant Rich Diet (PRD):** |  |  |  |  | 0.002 |
| No | 485 (23.4%) | 311 (21.3%) | 165 (28.7%) | 9 (24.3%) |  |
| Yes | 1590 (76.6%) | 1152 (78.7%) | 410 (71.3%) | 28 (75.7%) |  |
| **Western Diet (WD):** |  |  |  |  | 0.031 |
| No | 1842 (88.8%) | 1302 (89.0%) | 503 (87.5%) | 37 (100.0%) |  |
| Yes | 233 (11.2%) | 161 (11.0%) | 72 (12.5%) | 0 (0.0%) |  |
| **Self-perceived healthy eating habits (range 0 to 10)** | 6.9 (2.0) | 6.9 (2.0) | 6.9 (1.9) | 7.1 (2.0) | 0.788 |
| **Meal context:** |  |  |  |  | 0.661 |
| Alone | 707 (34.1%) | 491 (33.6%) | 200 (34.8%) | 16 (43.2%) |  |
| Distracted | 387 (18.7%) | 269 (18.4%) | 111 (19.3%) | 7 (18.9%) |  |
| Social | 981 (47.3%) | 703 (48.1%) | 264 (45.9%) | 14 (37.8%) |  |
| **Meals with others** |  |  |  |  | 0.017 |
| High | 1169 (56.3%) | 831 (56.8%) | 316 (55.0%) | 22 (59.5%) |  |
| Low | 119 (5.7%) | 68 (4.6%) | 47 (8.2%) | 4 (10.8%) |  |
| Medium | 787 (37.9%) | 564 (38.6%) | 212 (36.9%) | 11 (29.7%) |  |
| **Physical Activity (PA) level:** |  |  |  |  | 0.002 |
| Low | 496 (23.9%) | 371 (25.4%) | 120 (20.9%) | 5 (13.5%) |  |
| Moderate | 1190 (57.3%) | 843 (57.6%) | 320 (55.7%) | 27 (73.0%) |  |
| High | 389 (18.7%) | 249 (17.0%) | 135 (23.5%) | 5 (13.5%) |  |
| **Sitting hours per day** | 7.4 (3.3) | 7.3 (3.3) | 7.6 (3.3) | 8.1 (3.5) | 0.076 |
| **Sedentary behavior (SB):** |  |  |  |  | 0.235 |
| Active | 1305 (62.9%) | 935 (63.9%) | 350 (60.9%) | 20 (54.1%) |  |
| Sedentary | 770 (37.1%) | 528 (36.1%) | 225 (39.1%) | 17 (45.9%) |  |
| **Transport mode:** |  |  |  |  | . |
| Walk | 465 (22.4%) | 339 (23.2%) | 117 (20.3%) | 9 (24.3%) |  |
| Public Transport | 425 (20.5%) | 320 (21.9%) | 96 (16.7%) | 9 (24.3%) |  |
| Car or Motorbike | 1073 (51.7%) | 741 (50.6%) | 317 (55.1%) | 15 (40.5%) |  |
| Bike & Others | 112 (5.4%) | 63 (4.3%) | 45 (7.8%) | 4 (10.8%) |  |
| **Alcohol consumption:** |  |  |  |  | < 0.001 |
| Never | 488 (23.5%) | 369 (25.2%) | 112 (19.5%) | 7 (18.9%) |  |
| Almost Never | 552 (26.6%) | 416 (28.4%) | 127 (22.1%) | 9 (24.3%) |  |
| Occasionally | 652 (31.4%) | 464 (31.7%) | 177 (30.8%) | 11 (29.7%) |  |
| Weekly | 383 (18.5%) | 214 (14.6%) | 159 (27.7%) | 10 (27.0%) |  |
| **Binge drinking:** |  |  |  |  | 0.001 |
| Frequent | 29 (1.8%) | 14 (1.3%) | 15 (3.2%) | 0 (0.0%) |  |
| Never | 1119 (70.5%) | 803 (73.4%) | 299 (64.6%) | 17 (56.7%) |  |
| Occasional | 439 (27.7%) | 277 (25.3%) | 149 (32.2%) | 13 (43.3%) |  |
| **Alcohol quantity:** |  |  |  |  | . |
| ≥5 drinks | 73 (3.5%) | 38 (2.6%) | 34 (5.9%) | 1 (2.7%) |  |
| 1–2 drinks | 1192 (57.4%) | 824 (56.3%) | 345 (60.0%) | 23 (62.2%) |  |
| 3-4 drinks | 322 (15.5%) | 232 (15.9%) | 84 (14.6%) | 6 (16.2%) |  |
| Don't drink | 488 (23.5%) | 369 (25.2%) | 112 (19.5%) | 7 (18.9%) |  |
| **Smoking status:** |  |  |  |  | 0.167 |
| Non-smoker | 1638 (78.9%) | 1140 (77.9%) | 471 (81.9%) | 27 (73.0%) |  |
| Occasional smoker | 192 (9.3%) | 146 (10.0%) | 41 (7.1%) | 5 (13.5%) |  |
| Regular smoker | 245 (11.8%) | 177 (12.1%) | 63 (11.0%) | 5 (13.5%) |  |
| **Coffee consumption:** |  |  |  |  | 0.996 |
| High | 439 (21.2%) | 311 (21.3%) | 120 (20.9%) | 8 (21.6%) |  |
| Low | 388 (18.7%) | 274 (18.7%) | 108 (18.8%) | 6 (16.2%) |  |
| Moderate | 1248 (60.1%) | 878 (60.0%) | 347 (60.3%) | 23 (62.2%) |  |
| **Energy drink consumption:** |  |  |  |  | 0.31 |
| High | 4 (0.2%) | 3 (0.2%) | 1 (0.2%) | 0 (0.0%) |  |
| Low | 1981 (95.5%) | 1404 (96.0%) | 541 (94.1%) | 36 (97.3%) |  |
| Moderate | 90 (4.3%) | 56 (3.8%) | 33 (5.7%) | 1 (2.7%) |  |
| **Substance use (past month):** |  |  |  |  | 0.051 |
| No | 1921 (92.6%) | 1366 (93.4%) | 523 (91.0%) | 32 (86.5%) |  |
| Yes | 154 (7.4%) | 97 (6.6%) | 52 (9.0%) | 5 (13.5%) |  |
| **Mental well-being (GHQ-12):** |  |  |  |  | < 0.001 |
| Good | 286 (13.8%) | 199 (13.6%) | 85 (14.8%) | 2 (5.4%) |  |
| Moderate | 966 (46.6%) | 635 (43.4%) | 313 (54.4%) | 18 (48.6%) |  |
| Poor | 823 (39.7%) | 629 (43.0%) | 177 (30.8%) | 17 (45.9%) |  |
| **Satisfaction with relationships:** |  |  |  |  | 0.005 |
| High | 1476 (71.3%) | 1068 (73.1%) | 389 (68.1%) | 19 (51.4%) |  |
| Low | 53 (2.6%) | 31 (2.1%) | 21 (3.7%) | 1 (2.7%) |  |
| Medium | 541 (26.1%) | 363 (24.8%) | 161 (28.2%) | 17 (45.9%) |  |

**Note:** Values are presented as mean (SD) for continuous variables and n (%) for categorical variables. *p.overall* corresponds to global p-values obtained from comparison tests across gender categories (Male, Female, Others). *Mental health condition*: binary indicator for having any of the mental conditions (stress, anxiety, depression); *Migraine (chronic):* binary indicator of self-reported diagnosis of migraine (yes/no) categorized as part of chronic conditions; *Any disability* (including one of the following: vision, hearing, mobility, physical function, daily activities, assistive devices); *Meals with others:* High (≥5/week), Medium (2–4/week), and Low (<2/week) frequency of shared meals (breakfast, lunch, dinner); *Binge drinking:* Never, Occasional (monthly or less), and Frequent (weekly or more) for consuming ≥ 6 alcoholic units in one occasion; *Coffee and energy drink consumption:* Low (≤1 time/week), Moderate (2–6 times/week or once/day), and High (≥2 times/day); *Satisfaction with relationships:* Low (dissatisfied), Medium (acceptable), and High (satisfied) based on satisfaction with partner, family, or friends (5-point scale). **Test:** Continuous variables were tested using Kruskal-Wallis test, and categorical variables using chi-squared (χ2) or Fisher's exact test, as appropriate. The column *p value overall* corresponds to the global p-value for differences across gender groups for each variable. Dots (".") indicate that the p-value is not shown in this view but can be retrieved from contrast-specific comparisons or summary outputs in R.

1. **Staff**

|  | **[ALL]**  **N=533** | **Female N=370** | **Male N=159** | **Others N=4** | **p value overall** |
| --- | --- | --- | --- | --- | --- |
| **Age** | 44.5 (9.2) | 44.2 (8.7) | 45.3 (10.3) | 39.8 (5.2) | 0.241 |
| **Body Mass Index (BMI)** | 23.8 (3.7) | 23.4 (3.8) | 24.7 (3.3) | 21.9 (0.9) | <0.001 |
| **Work type:** |  |  |  |  | 0.226 |
| PAS | 305 (57.2%) | 218 (58.9%) | 85 (53.5%) | 2 (50.0%) |  |
| PDI | 116 (21.8%) | 72 (19.5%) | 42 (26.4%) | 2 (50.0%) |  |
| PDC | 112 (21.0%) | 80 (21.6%) | 32 (20.1%) | 0 (0.0%) |  |
| **Caregiving status:** |  |  |  |  | 0.008 |
| None | 256 (48.0%) | 162 (43.8%) | 91 (57.2%) | 3 (75.0%) |  |
| Children only | 242 (45.4%) | 183 (49.5%) | 59 (37.1%) | 0 (0.0%) |  |
| Dependents only | 17 (3.2%) | 11 (3.0%) | 6 (3.8%) | 0 (0.0%) |  |
| Children and dependents | 18 (3.4%) | 14 (3.8%) | 3 (1.9%) | 1 (25.0%) |  |
| **Birth place:** |  |  |  |  | 0.138 |
| Catalonia | 398 (74.7%) | 285 (77.0%) | 110 (69.2%) | 3 (75.0%) |  |
| Spain or Other | 135 (25.3%) | 85 (23.0%) | 49 (30.8%) | 1 (25.0%) |  |
| **Self-perceived socioeconomic status:** |  |  |  |  | 0.738 |
| Worse | 29 (5.4%) | 21 (5.7%) | 8 (5.0%) | 0 (0.0%) |  |
| Average | 343 (64.4%) | 243 (65.7%) | 97 (61.0%) | 3 (75.0%) |  |
| Better | 161 (30.2%) | 106 (28.6%) | 54 (34.0%) | 1 (25.0%) |  |
| **Education level:** |  |  |  |  | 0.625 |
| Low | 16 (3.0%) | 9 (2.4%) | 7 (4.4%) | 0 (0.0%) |  |
| Medium | 55 (10.3%) | 37 (10.0%) | 18 (11.3%) | 0 (0.0%) |  |
| High | 462 (86.7%) | 324 (87.6%) | 134 (84.3%) | 4 (100.0%) |  |
| **Occupational physical activity** |  |  |  |  | 0.041 |
| Low | 412 (79.1%) | 282 (78.6%) | 129 (81.6%) | 1 (25.0%) |  |
| Moderate | 98 (18.8%) | 71 (19.8%) | 24 (15.2%) | 3 (75.0%) |  |
| High | 11 (2.1%) | 6 (1.7%) | 5 (3.2%) | 0 (0.0%) |  |
| **Self-perceived health:** |  |  |  |  | 0.336 |
| Regular | 39 (7.3%) | 29 (7.8%) | 10 (6.3%) | 0 (0.0%) |  |
| Good | 244 (45.8%) | 168 (45.4%) | 72 (45.3%) | 4 (100.0%) |  |
| Very Good | 250 (46.9%) | 173 (46.8%) | 77 (48.4%) | 0 (0.0%) |  |
| **Sleeping hours per day** | 7.2 (0.8) | 7.2 (0.8) | 7.1 (0.8) | 7.2 (0.7) | 0.059 |
| **Sleep quality:** |  |  |  |  | 0.385 |
| Regular or Bad | 270 (50.7%) | 191 (51.6%) | 75 (47.2%) | 4 (100.0%) |  |
| Good | 53 (9.9%) | 36 (9.7%) | 17 (10.7%) | 0 (0.0%) |  |
| Very Good | 210 (39.4%) | 143 (38.6%) | 67 (42.1%) | 0 (0.0%) |  |
| **Mental health condition:** |  |  |  |  | 0.152 |
| No | 473 (88.7%) | 324 (87.6%) | 146 (91.8%) | 3 (75.0%) |  |
| Yes | 60 (11.3%) | 46 (12.4%) | 13 (8.2%) | 1 (25.0%) |  |
| **Migraine (chronic):** |  |  |  |  | 0.11 |
| No | 491 (92.1%) | 335 (90.5%) | 152 (95.6%) | 4 (100.0%) |  |
| Yes | 42 (7.9%) | 35 (9.5%) | 7 (4.4%) | 0 (0.0%) |  |
| **Any disability:** |  |  |  |  | 0.654 |
| No | 507 (96.8%) | 351 (97.0%) | 152 (96.2%) | 4 (100.0%) |  |
| Yes | 17 (3.2%) | 11 (3.0%) | 6 (3.8%) | 0 (0.0%) |  |
| **HEASUS index (range -1 to 10)** | 6.3 (1.0) | 6.4 (0.9) | 6.0 (1.0) | 5.9 (1.1) | <0.001 |
| **Plant Rich Diet (PRD):** |  |  |  |  | 0.02 |
| No | 116 (21.8%) | 69 (18.6%) | 46 (28.9%) | 1 (25.0%) |  |
| Yes | 417 (78.2%) | 301 (81.4%) | 113 (71.1%) | 3 (75.0%) |  |
| **Western Diet (WD):** |  |  |  |  | 0.44 |
| No | 497 (93.2%) | 348 (94.1%) | 145 (91.2%) | 4 (100.0%) |  |
| Yes | 36 (6.8%) | 22 (5.9%) | 14 (8.8%) | 0 (0.0%) |  |
| **Self-perceived healthy eating habits (range 0 to 10)** | 7.2 (1.7) | 7.3 (1.6) | 6.9 (1.7) | 6.5 (2.1) | 0.061 |
| **Meal context:** |  |  |  |  | 0.24 |
| Alone | 66 (12.4%) | 47 (12.7%) | 18 (11.3%) | 1 (25.0%) |  |
| Distracted | 228 (42.8%) | 162 (43.8%) | 63 (39.6%) | 3 (75.0%) |  |
| Social | 239 (44.8%) | 161 (43.5%) | 78 (49.1%) | 0 (0.0%) |  |
| **Meals with others:** |  |  |  |  | 0.576 |
| High | 227 (42.7%) | 162 (43.8%) | 63 (39.9%) | 2 (50.0%) |  |
| Low | 54 (10.2%) | 37 (10.0%) | 16 (10.1%) | 1 (25.0%) |  |
| Medium | 251 (47.2%) | 171 (46.2%) | 79 (50.0%) | 1 (25.0%) |  |
| **Physical Activity (PA) level:** |  |  |  |  | 0.088 |
| Low | 77 (14.4%) | 57 (15.4%) | 20 (12.6%) | 0 (0.0%) |  |
| Moderate | 386 (72.4%) | 272 (73.5%) | 112 (70.4%) | 2 (50.0%) |  |
| High | 70 (13.1%) | 41 (11.1%) | 27 (17.0%) | 2 (50.0%) |  |
| **Sitting hours per day** | 8.1 (2.8) | 8.1 (2.8) | 8.2 (2.8) | 7.2 (5.7) | 0.803 |
| **Sedentary behavior (SB):** |  |  |  |  | 0.331 |
| Active | 270 (50.7%) | 180 (48.6%) | 88 (55.3%) | 2 (50.0%) |  |
| Sedentary | 263 (49.3%) | 190 (51.4%) | 71 (44.7%) | 2 (50.0%) |  |
| **Transport mode:** |  |  |  |  | 0.582 |
| Walk | 190 (35.6%) | 139 (37.6%) | 50 (31.4%) | 1 (25.0%) |  |
| Public transport | 128 (24.0%) | 83 (22.4%) | 44 (27.7%) | 1 (25.0%) |  |
| Car or Motorbike | 166 (31.1%) | 115 (31.1%) | 50 (31.4%) | 1 (25.0%) |  |
| Bike & Others | 49 (9.2%) | 33 (8.9%) | 15 (9.4%) | 1 (25.0%) |  |
| **Alcohol consumption:** |  |  |  |  | <0.001 |
| Never | 76 (14.3%) | 65 (17.6%) | 11 (6.9%) | 0 (0.0%) |  |
| Almost Never | 92 (17.3%) | 70 (18.9%) | 21 (13.2%) | 1 (25.0%) |  |
| Occasionally | 206 (38.6%) | 142 (38.4%) | 61 (38.4%) | 3 (75.0%) |  |
| Weekly | 159 (29.8%) | 93 (25.1%) | 66 (41.5%) | 0 (0.0%) |  |
| **Binge drinking::** |  |  |  |  | 0.001 |
| Frequent | 2 (0.4%) | 1 (0.3%) | 1 (0.7%) | 0 (0.0%) |  |
| Never | 355 (77.7%) | 253 (83.0%) | 100 (67.6%) | 2 (50.0%) |  |
| Occasional | 100 (21.9%) | 51 (16.7%) | 47 (31.8%) | 2 (50.0%) |  |
| **Alcohol quantity:** |  |  |  |  | 0.001 |
| ≥5 drinks | 7 (1.5%) | 1 (0.3%) | 5 (3.4%) | 1 (25.0%) |  |
| 1–2 drinks | 389 (85.1%) | 270 (88.5%) | 116 (78.4%) | 3 (75.0%) |  |
| 3-4 drinks | 61 (13.3%) | 34 (11.1%) | 27 (18.2%) | 0 (0.0%) |  |
| **Smoking status:** |  |  |  |  | 0.015 |
| Non-smoker | 422 (79.2%) | 302 (81.6%) | 119 (74.8%) | 1 (25.0%) |  |
| Occasional smoker | 51 (9.6%) | 33 (8.9%) | 16 (10.1%) | 2 (50.0%) |  |
| Regular smoker | 60 (11.3%) | 35 (9.5%) | 24 (15.1%) | 1 (25.0%) |  |
| **Coffee consumption:** |  |  |  |  | 0.905 |
| High | 118 (22.1%) | 82 (22.2%) | 36 (22.6%) | 0 (0.0%) |  |
| Low | 79 (14.8%) | 55 (14.9%) | 23 (14.5%) | 1 (25.0%) |  |
| Moderate | 336 (63.0%) | 233 (63.0%) | 100 (62.9%) | 3 (75.0%) |  |
| **Energy drink consumption:** |  |  |  |  | 0.337 |
| High | 1 (0.2%) | 0 (0.0%) | 1 (0.6%) | 0 (0.0%) |  |
| Low | 527 (98.9%) | 367 (99.2%) | 156 (98.1%) | 4 (100.0%) |  |
| Moderate | 5 (0.9%) | 3 (0.8%) | 2 (1.3%) | 0 (0.0%) |  |
| **Substance use (past month):** |  |  |  |  | 0.007 |
| No | 506 (94.9%) | 358 (96.8%) | 145 (91.2%) | 3 (75.0%) |  |
| Yes | 27 (5.1%) | 12 (3.2%) | 14 (8.8%) | 1 (25.0%) |  |
| **Mental well-being (GHQ-12):** |  |  |  |  | 0.664 |
| Good | 51 (9.6%) | 37 (10.0%) | 14 (8.8%) | 0 (0.0%) |  |
| Moderate | 340 (63.8%) | 231 (62.4%) | 107 (67.3%) | 2 (50.0%) |  |
| Poor | 142 (26.6%) | 102 (27.6%) | 38 (23.9%) | 2 (50.0%) |  |
| **Satisfaction with relationships:** |  |  |  |  | 0.424 |
| High | 449 (84.4%) | 316 (85.6%) | 130 (81.8%) | 3 (75.0%) |  |
| Low | 5 (0.9%) | 4 (1.1%) | 1 (0.6%) | 0 (0.0%) |  |
| Medium | 78 (14.7%) | 49 (13.3%) | 28 (17.6%) | 1 (25.0%) |  |

**Note:** Values are presented as mean (SD) for continuous variables and n (%) for categorical variables. *p value overall* corresponds to global p-values obtained from comparison tests across gender categories (Male, Female, Others). *Work type*: PAS (Administrative and Service Staff), PDI (Teaching and Research Staff), and PDC (Administrative and Service Staff). *Education level*: Low (Secondary or High School, including “Other”), Medium (Vocational training or first university cycle), and High (Second university cycle/postgraduate education); *Occupational PA*: Low (mostly sitting or standing), Moderate (frequent walking), and High (physically demanding activity); *Mental health condition*: binary indicator for having any of the mental conditions (stress, anxiety, depression); *Migraine (chronic):* binary indicator of self-reported diagnosis of migraine (yes/no) categorized as part of chronic conditions; *Any disability* (including one of the following: vision, hearing, mobility, physical function, daily activities, assistive devices); *Meals with others:* High (≥5/week), Medium (2–4/week), and Low (<2/week) frequency of shared meals (breakfast, lunch, dinner); *Binge drinking:* Never, Occasional (monthly or less), and Frequent (weekly or more) for consuming ≥ 6 alcoholic units in one occasion; *Coffee and energy drink consumption:* Low (≤1 time/week), Moderate (2–6 times/week or once/day), and High (≥2 times/day); *Satisfaction with relationships:* Low (dissatisfied), Medium (acceptable), and High (satisfied) based on satisfaction with partner, family, or friends (5-point scale). **Test:** Continuous variables were tested using Kruskal-Wallis test, and categorical variables using chi-squared (χ2) or Fisher's exact test, as appropriate. The column *p value overall* corresponds to the global p-value for differences across gender groups for each variable. Dots (".") indicate that the p-value is not shown in this view but can be retrieved from contrast-specific comparisons or summary outputs in R.

**Supplementary Table 2: Health, lifestyle, and dietary patterns by physical activity (PA) level.**

1. **Students**

|  | **Low**  **N=491** | **Moderate**  **N=1163** | **High**  **N=384** | ***P* value overall** |
| --- | --- | --- | --- | --- |
| **Gender:** |  |  |  | 0.002 |
| Female | 371 (75.6%) | 843 (72.5%) | 249 (64.8%) |  |
| Male | 120 (24.4%) | 320 (27.5%) | 135 (35.2%) |  |
| **Age** | 35.7 (10.2) | 36.0 (10.7) | 33.3 (10.5) | <0.001 |
| **Body Mass Index (BMI)** | 24.6 (5.0) | 23.7 (4.1) | 23.2 (3.6) | <0.001 |
| **Student type:** |  |  |  | 0.202 |
| Undergraduate | 313 (63.7%) | 724 (62.3%) | 264 (68.8%) |  |
| PhD | 12 (2.4%) | 38 (3.3%) | 9 (2.3%) |  |
| Master/postgraduate | 166 (33.8%) | 401 (34.5%) | 111 (28.9%) |  |
| **Type of study:** |  |  |  | 0.797 |
| Arts/Humanities | 104 (21.2%) | 233 (20.0%) | 71 (18.5%) |  |
| Engineering | 90 (18.3%) | 193 (16.6%) | 64 (16.7%) |  |
| Health | 48 (9.8%) | 121 (10.4%) | 47 (12.2%) |  |
| Social/Legal | 249 (50.7%) | 616 (53.0%) | 202 (52.6%) |  |
| **Place of residence:** |  |  |  | 0.95 |
| Catalonia | 294 (59.9%) | 701 (60.3%) | 228 (59.4%) |  |
| Spain or Other | 197 (40.1%) | 462 (39.7%) | 156 (40.6%) |  |
| **Household Composition:** |  |  |  | 0.023 |
| Family or Partner | 421 (85.7%) | 935 (80.4%) | 304 (79.2%) |  |
| Shared Accommodation | 9 (1.8%) | 60 (5.2%) | 16 (4.2%) |  |
| Alone | 43 (8.8%) | 131 (11.3%) | 49 (12.8%) |  |
| Other Relatives and Others | 18 (3.7%) | 37 (3.2%) | 15 (3.9%) |  |
| **Work status:** |  |  |  | 0.453 |
| Unemployed | 108 (22.0%) | 215 (18.5%) | 67 (17.4%) |  |
| Part Time | 85 (17.3%) | 212 (18.2%) | 69 (18.0%) |  |
| Full Time | 298 (60.7%) | 736 (63.3%) | 248 (64.6%) |  |
| **Self-perceived health:** |  |  |  | <0.001 |
| Regular or Bad | 115 (23.4%) | 140 (12.0%) | 37 (9.6%) |  |
| Good | 229 (46.6%) | 480 (41.3%) | 114 (29.7%) |  |
| Very Good | 147 (29.9%) | 543 (46.7%) | 233 (60.7%) |  |
| **Sleeping hours per day** | 7.0 (1.1) | 7.1 (1.0) | 7.2 (1.0) | 0.001 |
| **Sleep quality:** |  |  |  | <0.001 |
| Regular or Bad | 267 (54.4%) | 490 (42.1%) | 136 (35.4%) |  |
| Good | 131 (26.7%) | 397 (34.1%) | 128 (33.3%) |  |
| Very Good | 93 (18.9%) | 276 (23.7%) | 120 (31.2%) |  |
| **Mental health condition:** |  |  |  | <0.001 |
| No | 180 (36.7%) | 574 (49.4%) | 200 (52.1%) |  |
| Yes | 311 (63.3%) | 589 (50.6%) | 184 (47.9%) |  |
| **Migraine (chronic):** |  |  |  | 0.067 |
| No | 351 (71.5%) | 864 (74.3%) | 301 (78.4%) |  |
| Yes | 140 (28.5%) | 299 (25.7%) | 83 (21.6%) |  |
| **Any disability:** |  |  |  | <0.001 |
| No | 419 (87.8%) | 1061 (93.2%) | 360 (94.5%) |  |
| Yes | 58 (12.2%) | 78 (6.8%) | 21 (5.5%) |  |
| **HEASUS index (range -1 to 10)** | 5.6 (1.1) | 6.1 (1.1) | 6.3 (1.1) | <0.001 |
| **Plant Rich Diet (PRD):** |  |  |  | <0.001 |
| No | 160 (32.6%) | 229 (19.7%) | 87 (22.7%) |  |
| Yes | 331 (67.4%) | 934 (80.3%) | 297 (77.3%) |  |
| **Western Diet (WD):** |  |  |  | 0.776 |
| No | 438 (89.2%) | 1025 (88.1%) | 342 (89.1%) |  |
| Yes | 53 (10.8%) | 138 (11.9%) | 42 (10.9%) |  |
| **Self-perceived healthy eating habits (range 0 to 10)** | 6.2 (2.1) | 7.0 (1.8) | 7.5 (2.0) | <0.001 |
| **Meal context:** |  |  |  | 0.193 |
| Alone | 163 (33.2%) | 402 (34.6%) | 126 (32.8%) |  |
| Distracted | 94 (19.1%) | 199 (17.1%) | 87 (22.7%) |  |
| Social | 234 (47.7%) | 562 (48.3%) | 171 (44.5%) |  |
| **Meals with others:** |  |  |  | 0.227 |
| High | 275 (56.0%) | 670 (57.6%) | 202 (52.6%) |  |
| Low | 34 (6.9%) | 56 (4.8%) | 25 (6.5%) |  |
| Medium | 182 (37.1%) | 437 (37.6%) | 157 (40.9%) |  |
| **Sitting hours per day** | 8.6 (3.4) | 7.2 (3.2) | 6.2 (3.0) | <0.001 |
| **Sedentary behavior (SB):** |  |  |  | <0.001 |
| Active | 230 (46.8%) | 753 (64.7%) | 302 (78.6%) |  |
| Sedentary | 261 (53.2%) | 410 (35.3%) | 82 (21.4%) |  |
| **Transport mode:** |  |  |  | <0.001 |
| Walk | 90 (18.3%) | 258 (22.2%) | 108 (28.1%) |  |
| Public Transport | 100 (20.4%) | 239 (20.6%) | 77 (20.1%) |  |
| Car or Motorbike | 289 (58.9%) | 604 (51.9%) | 165 (43.0%) |  |
| Bike & Others | 12 (2.4%) | 62 (5.3%) | 34 (8.9%) |  |
| **Alcohol consumption:** |  |  |  | 0.008 |
| Never | 144 (29.3%) | 250 (21.5%) | 87 (22.7%) |  |
| Almost Never | 130 (26.5%) | 322 (27.7%) | 91 (23.7%) |  |
| Occasionally | 132 (26.9%) | 371 (31.9%) | 138 (35.9%) |  |
| Weekly | 85 (17.3%) | 220 (18.9%) | 68 (17.7%) |  |
| **Binge drinking:** |  |  |  | 0.167 |
| Frequent | 6 (1.7%) | 14 (1.5%) | 9 (3.0%) |  |
| Never | 241 (69.5%) | 664 (72.7%) | 197 (66.3%) |  |
| Occasional | 100 (28.8%) | 235 (25.7%) | 91 (30.6%) |  |
| **Alcohol quantity:** |  |  |  | 0.006 |
| ≥5 drinks | 14 (2.9%) | 38 (3.3%) | 20 (5.2%) |  |
| 1–2 drinks | 263 (53.6%) | 697 (59.9%) | 209 (54.4%) |  |
| 3-4 drinks | 70 (14.3%) | 178 (15.3%) | 68 (17.7%) |  |
| Don't drink | 144 (29.3%) | 250 (21.5%) | 87 (22.7%) |  |
| **Smoking status:** |  |  |  | 0.075 |
| Non-smoker | 376 (76.6%) | 914 (78.6%) | 321 (83.6%) |  |
| Occasional smoker | 46 (9.4%) | 109 (9.4%) | 32 (8.3%) |  |
| Regular smoker | 69 (14.1%) | 140 (12.0%) | 31 (8.1%) |  |
| **Coffee consumption:** |  |  |  | 0.877 |
| High | 99 (20.2%) | 245 (21.1%) | 87 (22.7%) |  |
| Low | 92 (18.7%) | 223 (19.2%) | 67 (17.4%) |  |
| Moderate | 300 (61.1%) | 695 (59.8%) | 230 (59.9%) |  |
| **Energy drink consumption:** |  |  |  | 0.502 |
| High | 1 (0.2%) | 3 (0.3%) | 0 (0.0%) |  |
| Low | 468 (95.3%) | 1115 (95.9%) | 362 (94.3%) |  |
| Moderate | 22 (4.5%) | 45 (3.9%) | 22 (5.7%) |  |
| **Substance use (past month):** |  |  |  | 0.972 |
| No | 455 (92.7%) | 1077 (92.6%) | 357 (93.0%) |  |
| Yes | 36 (7.3%) | 86 (7.4%) | 27 (7.0%) |  |
| **Emotional well-being (GHQ-12):** |  |  |  | <0.001 |
| Good | 52 (10.6%) | 166 (14.3%) | 66 (17.2%) |  |
| Moderate | 196 (39.9%) | 557 (47.9%) | 195 (50.8%) |  |
| Poor | 243 (49.5%) | 440 (37.8%) | 123 (32.0%) |  |
| **Satisfaction with relationships:** |  |  |  | <0.001 |
| High | 312 (63.7%) | 848 (73.2%) | 297 (77.3%) |  |
| Low | 21 (4.3%) | 25 (2.2%) | 6 (1.6%) |  |
| Medium | 157 (32.0%) | 286 (24.7%) | 81 (21.1%) |  |

**Note:** Values are presented as mean (SD) for continuous variables and n (%) for categorical variables. *p.overall* corresponds to global p-values obtained from comparison tests across PA categories (Low, Moderate, High). *Mental health condition*: binary indicator for having any of the mental conditions (stress, anxiety, depression); *Migraine (chronic):* binary indicator of self-reported diagnosis of migraine (yes/no) categorized as part of chronic conditions; *Any disability* (including one of the following: vision, hearing, mobility, physical function, daily activities, assistive devices); *Meals with others*: High (≥5/week), Medium (2–4/week), and Low (<2/week) frequency of shared meals (breakfast, lunch, dinner)*; Binge drinking;* Never, Occasional (monthly or less), and Frequent (weekly or more) for consuming ≥ 6 alcoholic units in one occasion*; Coffee and energy drink consumption:* Low (≤1 time/week), Moderate (2–6 times/week or once/day), and High (≥2 times/day); *Satisfaction with relationships:* Low (dissatisfied), Medium (acceptable), and High (satisfied) based on satisfaction with partner, family, or friends (5-point scale). **Test:** Continuous variables were tested using Kruskal-Wallis test, and categorical variables using chi-squared (χ2) or Fisher's exact test, as appropriate. The column *p value overall* corresponds to the global p-value for differences across gender groups for each variable.

1. **Staff**

|  | **Low**  **N=77** | **Moderate**  **N=384** | **High**  **N=68** | ***P* value overall** |
| --- | --- | --- | --- | --- |
| **Gender:** |  |  |  | 0.152 |
| Female | 57 (74.0%) | 272 (70.8%) | 41 (60.3%) |  |
| Male | 20 (26.0%) | 112 (29.2%) | 27 (39.7%) |  |
| **Age** | 43.7 (8.8) | 44.6 (9.0) | 44.9 (10.6) | 0.67 |
| **Body Mass Index (BMI)** | 24.0 (4.0) | 23.7 (3.8) | 23.9 (3.4) | 0.83 |
| **Work type:** |  |  |  | 0.689 |
| PAS | 48 (62.3%) | 217 (56.5%) | 38 (55.9%) |  |
| PDI | 14 (18.2%) | 82 (21.4%) | 18 (26.5%) |  |
| PDC | 15 (19.5%) | 85 (22.1%) | 12 (17.6%) |  |
| **Caregiving status:** |  |  |  | 0.74 |
| None | 34 (44.2%) | 180 (46.9%) | 39 (57.4%) |  |
| Children only | 37 (48.1%) | 179 (46.6%) | 26 (38.2%) |  |
| Dependents only | 3 (3.9%) | 13 (3.4%) | 1 (1.5%) |  |
| Children and dependents | 3 (3.9%) | 12 (3.1%) | 2 (2.9%) |  |
| **Birth place:** |  |  |  | 0.205 |
| Catalonia | 62 (80.5%) | 287 (74.7%) | 46 (67.6%) |  |
| Spain or Other | 15 (19.5%) | 97 (25.3%) | 22 (32.4%) |  |
| **Self-perceived socioeconomic status:** |  |  |  | . |
| Worse | 8 (10.4%) | 19 (4.9%) | 2 (2.9%) |  |
| Average | 47 (61.0%) | 249 (64.8%) | 44 (64.7%) |  |
| Better | 22 (28.6%) | 116 (30.2%) | 22 (32.4%) |  |
| **Education level:** |  |  |  | 0.721 |
| Low | 3 (3.9%) | 10 (2.6%) | 3 (4.4%) |  |
| Medium | 6 (7.8%) | 43 (11.2%) | 6 (8.8%) |  |
| High | 68 (88.3%) | 331 (86.2%) | 59 (86.8%) |  |
| **Occupational physical activity:** |  |  |  | 0.025 |
| Low | 70 (90.9%) | 294 (78.6%) | 47 (71.2%) |  |
| Moderate | 7 (9.1%) | 72 (19.3%) | 16 (24.2%) |  |
| High | 0 (0.0%) | 8 (2.1%) | 3 (4.5%) |  |
| **Self-perceived health:** |  |  |  | <0.001 |
| Regular | 14 (18.2%) | 25 (6.5%) | 0 (0.0%) |  |
| Good | 38 (49.4%) | 171 (44.5%) | 31 (45.6%) |  |
| Very Good | 25 (32.5%) | 188 (49.0%) | 37 (54.4%) |  |
| **Sleeping hours per day** | 7.0 (0.9) | 7.2 (0.8) | 7.2 (0.7) | 0.192 |
| **Sleep quality:** |  |  |  | 0.006 |
| Regular or Bad | 52 (67.5%) | 185 (48.2%) | 29 (42.6%) |  |
| Good | 9 (11.7%) | 36 (9.4%) | 8 (11.8%) |  |
| Very Good | 16 (20.8%) | 163 (42.4%) | 31 (45.6%) |  |
| **Emotional health condition:** |  |  |  | 0.37 |
| No | 65 (84.4%) | 343 (89.3%) | 62 (91.2%) |  |
| Yes | 12 (15.6%) | 41 (10.7%) | 6 (8.8%) |  |
| **Migraine (chronic):** |  |  |  | 0.082 |
| No | 66 (85.7%) | 358 (93.2%) | 63 (92.6%) |  |
| Yes | 11 (14.3%) | 26 (6.8%) | 5 (7.4%) |  |
| **Any disability:** |  |  |  | 0.392 |
| No | 72 (97.3%) | 368 (97.1%) | 63 (94.0%) |  |
| Yes | 2 (2.7%) | 11 (2.9%) | 4 (6.0%) |  |
| **HEASUS index (range -1 to 10)** | 6.1 (0.8) | 6.3 (1.0) | 6.3 (1.0) | 0.062 |
| **Plant Rich Diet (PRD):** |  |  |  | 0.924 |
| No | 18 (23.4%) | 82 (21.4%) | 15 (22.1%) |  |
| Yes | 59 (76.6%) | 302 (78.6%) | 53 (77.9%) |  |
| **Western Diet (WD):** |  |  |  | 0.032 |
| No | 66 (85.7%) | 362 (94.3%) | 65 (95.6%) |  |
| Yes | 11 (14.3%) | 22 (5.7%) | 3 (4.4%) |  |
| **Self-perceived healthy eating habits (range 0 to 10)** | 6.7 (1.9) | 7.2 (1.6) | 7.6 (1.8) | 0.004 |
| **Meal context:** |  |  |  | 0.026 |
| Alone | 1 (1.3%) | 55 (14.3%) | 9 (13.2%) |  |
| Distracted | 37 (48.1%) | 156 (40.6%) | 32 (47.1%) |  |
| Social | 39 (50.6%) | 173 (45.1%) | 27 (39.7%) |  |
| **Meals with others:** |  |  |  | 0.412 |
| High | 38 (50.0%) | 159 (41.4%) | 28 (41.2%) |  |
| Low | 7 (9.2%) | 36 (9.4%) | 10 (14.7%) |  |
| Medium | 31 (40.8%) | 189 (49.2%) | 30 (44.1%) |  |
| **Sitting hours per day** | 8.3 (3.3) | 8.1 (2.7) | 8.1 (2.4) | 0.806 |
| **Sedentary behavior (SB):** |  |  |  | 0.614 |
| Active | 37 (48.1%) | 193 (50.3%) | 38 (55.9%) |  |
| Sedentary | 40 (51.9%) | 191 (49.7%) | 30 (44.1%) |  |
| **Transport mode:** |  |  |  | 0.284 |
| Walk | 21 (27.3%) | 140 (36.5%) | 28 (41.2%) |  |
| Public transport | 23 (29.9%) | 87 (22.7%) | 17 (25.0%) |  |
| Car or Motorbike | 29 (37.7%) | 120 (31.2%) | 16 (23.5%) |  |
| Bike & Others | 4 (5.2%) | 37 (9.6%) | 7 (10.3%) |  |
| **Alcohol consumption:** |  |  |  | 0.307 |
| Never | 10 (13.0%) | 57 (14.8%) | 9 (13.2%) |  |
| Almost Never | 19 (24.7%) | 57 (14.8%) | 15 (22.1%) |  |
| Occasionally | 31 (40.3%) | 148 (38.5%) | 24 (35.3%) |  |
| Weekly | 17 (22.1%) | 122 (31.8%) | 20 (29.4%) |  |
| **Alcohol quantity:** |  |  |  | 0.048 |
| one or two | 64 (95.5%) | 274 (83.8%) | 48 (81.4%) |  |
| Three or four | 3 (4.5%) | 49 (15.0%) | 9 (15.3%) |  |
| Five or six | 0 (0.0%) | 4 (1.2%) | 2 (3.4%) |  |
| **Binge drinking:** |  |  |  | 0.25 |
| Frequent | 0 (0.0%) | 2 (0.6%) | 0 (0.0%) |  |
| Never | 55 (82.1%) | 258 (78.9%) | 40 (67.8%) |  |
| Occasional | 12 (17.9%) | 67 (20.5%) | 19 (32.2%) |  |
| **Alcohol quantity:** |  |  |  | 0.048 |
| ≥5 drinks | 0 (0.0%) | 4 (1.2%) | 2 (3.4%) |  |
| 1–2 drinks | 64 (95.5%) | 274 (83.8%) | 48 (81.4%) |  |
| 3-4 drinks | 3 (4.5%) | 49 (15.0%) | 9 (15.3%) |  |
| **Smoking status:** |  |  |  | 0.473 |
| Non-smoker | 58 (75.3%) | 308 (80.2%) | 55 (80.9%) |  |
| Occasional smoker | 11 (14.3%) | 34 (8.9%) | 4 (5.9%) |  |
| Regular smoker | 8 (10.4%) | 42 (10.9%) | 9 (13.2%) |  |
| **Coffee consumption:** |  |  |  | 0.721 |
| High | 16 (20.8%) | 85 (22.1%) | 17 (25.0%) |  |
| Low | 15 (19.5%) | 55 (14.3%) | 8 (11.8%) |  |
| Moderate | 46 (59.7%) | 244 (63.5%) | 43 (63.2%) |  |
| **Energy drink consumption:** |  |  |  | 1 |
| High | 0 (0.0%) | 1 (0.3%) | 0 (0.0%) |  |
| Low | 76 (98.7%) | 379 (98.7%) | 68 (100.0%) |  |
| Moderate | 1 (1.3%) | 4 (1.0%) | 0 (0.0%) |  |
| **Substance use (past month):** |  |  |  | 0.019 |
| No | 71 (92.2%) | 371 (96.6%) | 61 (89.7%) |  |
| Yes | 6 (7.8%) | 13 (3.4%) | 7 (10.3%) |  |
| **Emotional well-being (GHQ-12):** |  |  |  | 0.004 |
| Good | 2 (2.6%) | 41 (10.7%) | 8 (11.8%) |  |
| Moderate | 42 (54.5%) | 250 (65.1%) | 46 (67.6%) |  |
| Poor | 33 (42.9%) | 93 (24.2%) | 14 (20.6%) |  |
| **Satisfaction with relationships:** |  |  |  | 0.112 |
| High | 58 (75.3%) | 330 (86.2%) | 58 (85.3%) |  |
| Low | 2 (2.6%) | 3 (0.8%) | 0 (0.0%) |  |
| Medium | 17 (22.1%) | 50 (13.1%) | 10 (14.7%) |  |

**Note:** Values are presented as mean (SD) for continuous variables and n (%) for categorical variables. *p.overall* corresponds to global p-values obtained from comparison tests PA categories (Low, Moderate, High). *Work type:* PAS (Administrative and Service Staff), PDI (Teaching and Research Staff), and PDC (Administrative and Service Staff); *Education level*: Low (Secondary or High School, including “Other”), Medium (Vocational training or first university cycle), and High (second cycle/postgraduate education); *Occupational PA*: Low (mostly sitting or standing), Moderate (frequent walking), and High (physically demanding activity); *Mental health condition*: binary indicator for having any of the mental conditions (stress, anxiety, depression); *Migraine (chronic):* binary indicator of self-reported diagnosis of migraine (yes/no) categorized as part of chronic conditions; *Any disability* (including one of the following: vision, hearing, mobility, physical function, daily activities, assistive devices); *Meals with others:* High (≥5/week), Medium (2–4/week), and Low (<2/week) frequency of shared meals (breakfast, lunch, dinner); *Binge drinking:* Never, Occasional (monthly or less), and Frequent (weekly or more) for consuming ≥ 6 alcoholic units in one occasion; *Coffee and energy drink consumption:* Low (≤1 time/week), Moderate (2–6 times/week or once/day), and High (≥2 times/day); *Satisfaction with relationships:* Low (dissatisfied), Medium (acceptable), and High (satisfied) based on satisfaction with partner, family, or friends (5-point scale). **Test:** Continuous variables were tested using Kruskal-Wallis test, and categorical variables using chi-squared (χ2) or Fisher's exact test, as appropriate. The column *p value overall* corresponds to the global p-value for differences across gender groups for each variable

**Supplementary Table 3: Distribution of main variables across HEASUS tertiles adherence, separately calculated for each population group. *T1: Low adherence; T2: Medium adherence; T3: High adherence.***

1. **Students**

|  | **T1**  **(N=696)** | **T2**  **(N=695)** | **T3**  **(N=684)** | **[ALL]**  **(N=2075)** | ***p value overall*** |
| --- | --- | --- | --- | --- | --- |
| **HEASUS index range** | ≤ 5.56 | 5.57 - 6.54 | ≥ 6.55 |  |  |
| **HEASUS score (range -1 to 10)** | 4.8 (± 0.66) | 6.1 (± 0.28) | 7.2 (± 0.50) | 6.0 (± 1.1) | - |
| **Gender** |  |  |  |  | 0.014 |
| Male | 217 (31 %) | 200 (29 %) | 158 (23 %) | 575 (28 %) |  |
| Female | 469 (67 %) | 483 (69 %) | 511 (75 %) | 1463 (71 %) |  |
| Others | 10 (1 %) | 12 (2 %) | 15 (2 %) | 37 (2 %) |  |
| **Age** | 33 (± 9.7) | 36 (± 11) | 37 (± 11) | 35 (± 11) | < 0.001 |
| **Body Mass Index (BMI)** | 24 (± 4.7) | 24 (± 4.3) | 23 (± 3.7) | 24 (± 4.3) | 0.099 |
| **Student type** |  |  |  |  | < 0.001 |
| Undergraduate | 476 (68 %) | 458 (66 %) | 395 (58 %) | 1329 (64 %) |  |
| PhD | 20 (3 %) | 17 (2 %) | 23 (3 %) | 60 (3 %) |  |
| Master/postgraduate | 200 (29 %) | 220 (32 %) | 266 (39 %) | 686 (33 %) |  |
| **Type of study:** |  |  |  |  | < 0.001 |
| Arts/Humanities | 142 (20 %) | 131 (19 %) | 148 (22 %) | 421 (20 %) |  |
| Engineering | 137 (20 %) | 112 (16 %) | 104 (15 %) | 353 (17 %) |  |
| Health | 53 (8 %) | 58 (8 %) | 108 (16 %) | 219 (11 %) |  |
| Social/Legal | 364 (52 %) | 394 (57 %) | 324 (47 %) | 1082 (52 %) |  |
| **Place of residence:** |  |  |  |  | 0.21 |
| Catalonia | 436 (63 %) | 412 (59 %) | 398 (58 %) | 1246 (60 %) |  |
| Spain or Other | 260 (37 %) | 283 (41 %) | 286 (42 %) | 829 (40 %) |  |
| **Household Composition:** |  |  |  |  | 0.754 |
| Family or Partner | 565 (81 %) | 567 (82 %) | 553 (81 %) | 1685 (81 %) |  |
| Shared Accommodation | 34 (5 %) | 28 (4 %) | 27 (4 %) | 89 (4 %) |  |
| Alone | 68 (10 %) | 78 (11 %) | 81 (12 %) | 227 (11 %) |  |
| Other Relatives and Others | 29 (4 %) | 22 (3 %) | 23 (3 %) | 74 (4 %) |  |
| **Work status:** |  |  |  |  | 0.326 |
| Unemployed | 143 (21 %) | 132 (19 %) | 124 (18 %) | 399 (19 %) |  |
| Part Time | 131 (19 %) | 112 (16 %) | 134 (20 %) | 377 (18 %) |  |
| Full Time | 422 (61 %) | 451 (65 %) | 426 (62 %) | 1299 (63 %) |  |
| **Self-perceived health:** |  |  |  |  | < 0.001 |
| Regular or Bad | 138 (20 %) | 89 (13 %) | 75 (11 %) | 302 (15 %) |  |
| Good | 311 (45 %) | 279 (40 %) | 250 (37 %) | 840 (40 %) |  |
| Very Good | 247 (35 %) | 327 (47 %) | 359 (52 %) | 933 (45 %) |  |
| **Sleeping hours per day** | 7.1 (± 1.1) | 7.1 (± 1.0) | 7.1 (± 0.89) | 7.1 (± 1.0) | 0.013 |
| **Sleep quality:** |  |  |  |  | 0.017 |
| Regular or Bad | 332 (48 %) | 302 (43 %) | 274 (40 %) | 908 (44 %) |  |
| Good | 225 (32 %) | 222 (32 %) | 226 (33 %) | 673 (32 %) |  |
| Very Good | 139 (20 %) | 171 (25 %) | 184 (27 %) | 494 (24 %) |  |
| **Chronic mental health:** |  |  |  |  | < 0.001 |
| No | 275 (40 %) | 348 (50 %) | 344 (50 %) | 967 (47 %) |  |
| Yes | 421 (60 %) | 347 (50 %) | 340 (50 %) | 1108 (53 %) |  |
| **Migraine (chronic):** |  |  |  |  | < 0.001 |
| No | 480 (69 %) | 529 (76 %) | 534 (78 %) | 1543 (74 %) |  |
| Yes | 216 (31 %) | 166 (24 %) | 150 (22 %) | 532 (26 %) |  |
| **Any disability:** |  |  |  |  | 0.814 |
| No | 616 (89 %) | 639 (92 %) | 615 (90 %) | 1870 (90 %) |  |
| Yes | 55 (8 %) | 51 (7 %) | 55 (8 %) | 161 (8 %) |  |
| Missing | 25 (3.6%) | 5 (0.7%) | 14 (2.0%) | 44 (2.1%) |  |
| **Plant Rich Diet (PRD):** |  |  |  |  | < 0.001 |
| No | 280 (40 %) | 134 (19 %) | 71 (10 %) | 485 (23 %) |  |
| Yes | 416 (60 %) | 561 (81 %) | 613 (90 %) | 1590 (77 %) |  |
| **Western Diet (WD):** |  |  |  |  | < 0.001 |
| No | 570 (82 %) | 621 (89 %) | 651 (95 %) | 1842 (89 %) |  |
| Yes | 126 (18 %) | 74 (11 %) | 33 (5 %) | 233 (11 %) |  |
| **Self-perceived healthy eating habits (range 0 to 10)** | 5.8 (± 2.1) | 7.1 (± 1.7) | 7.9 (± 1.6) | 6.9 (± 2.0) | < 0.001 |
| **Meal context:** |  |  |  |  | 0.009 |
| Alone | 274 (39 %) | 222 (32 %) | 211 (31 %) | 707 (34 %) |  |
| Distracted | 121 (17 %) | 134 (19 %) | 132 (19 %) | 387 (19 %) |  |
| Social | 301 (43 %) | 339 (49 %) | 341 (50 %) | 981 (47 %) |  |
| **Meals with others:** |  |  |  |  | 0.029 |
| High | 366 (53 %) | 407 (59 %) | 396 (58 %) | 1169 (56 %) |  |
| Low | 51 (7 %) | 40 (6 %) | 28 (4 %) | 119 (6 %) |  |
| Medium | 279 (40 %) | 248 (36 %) | 260 (38 %) | 787 (38 %) |  |
| **Physical Activity (PA) level:** |  |  |  |  | < 0.001 |
| Low | 236 (34 %) | 155 (22 %) | 105 (15 %) | 496 (24 %) |  |
| Moderate | 361 (52 %) | 419 (60 %) | 410 (60 %) | 1190 (57 %) |  |
| High | 99 (14 %) | 121 (17 %) | 169 (25 %) | 389 (19 %) |  |
| **Sitting hours per day** | 7.8 (± 3.4) | 7.4 (± 3.2) | 7.0 (± 3.3) | 7.4 (± 3.3) | < 0.001 |
| **Sedentary behavior (SB):** |  |  |  |  | < 0.001 |
| Active | 398 (57 %) | 444 (64 %) | 463 (68 %) | 1305 (63 %) |  |
| Sedentary | 298 (43 %) | 251 (36 %) | 221 (32 %) | 770 (37 %) |  |
| **Transport mode:** |  |  |  |  | 0.021 |
| Walk | 138 (20 %) | 150 (22 %) | 177 (26 %) | 465 (22 %) |  |
| Public Transport | 150 (22 %) | 139 (20 %) | 136 (20 %) | 425 (20 %) |  |
| Car or Motorbike | 380 (55 %) | 368 (53 %) | 325 (48 %) | 1073 (52 %) |  |
| Bike & Others | 28 (4 %) | 38 (5 %) | 46 (7 %) | 112 (5 %) |  |
| **Alcohol consumption:** |  |  |  |  | 0.022 |
| Never | 174 (25 %) | 140 (20 %) | 174 (25 %) | 488 (24 %) |  |
| Almost Never | 203 (29 %) | 182 (26 %) | 167 (24 %) | 552 (27 %) |  |
| Occasionally | 198 (28 %) | 227 (33 %) | 227 (33 %) | 652 (31 %) |  |
| Weekly | 121 (17 %) | 146 (21 %) | 116 (17 %) | 383 (18 %) |  |
| **Binge drinking:** |  |  |  |  | 0.174 |
| Frequent | 14 (2 %) | 10 (1 %) | 5 (1 %) | 29 (1 %) |  |
| Never | 354 (51 %) | 392 (56 %) | 373 (55 %) | 1119 (54 %) |  |
| Occasional | 154 (22 %) | 153 (22 %) | 132 (19 %) | 439 (21 %) |  |
| Missing | 174 (25.0%) | 140 (20.1%) | 174 (25.4%) | 488 (23.5%) |  |
| **Alcohol quantity:** |  |  |  |  | 0.001 |
| ≥5 drinks | 35 (5 %) | 19 (3 %) | 19 (3 %) | 73 (4 %) |  |
| 1–2 drinks | 373 (54 %) | 412 (59 %) | 407 (60 %) | 1192 (57 %) |  |
| 3-4 drinks | 114 (16 %) | 124 (18 %) | 84 (12 %) | 322 (16 %) |  |
| Missing | 174 (25 %) | 140 (20 %) | 174 (25 %) | 488 (24 %) |  |
| **Smoking status:** |  |  |  |  | < 0.001 |
| Non-smoker | 521 (75 %) | 542 (78 %) | 575 (84 %) | 1638 (79 %) |  |
| Occasional smoker | 66 (9 %) | 64 (9 %) | 62 (9 %) | 192 (9 %) |  |
| Regular smoker | 109 (16 %) | 89 (13 %) | 47 (7 %) | 245 (12 %) |  |
| **Coffee consumption:** |  |  |  |  | < 0.001 |
| High | 134 (19 %) | 114 (16 %) | 191 (28 %) | 439 (21 %) |  |
| Low | 170 (24 %) | 113 (16 %) | 105 (15 %) | 388 (19 %) |  |
| Moderate | 392 (56 %) | 468 (67 %) | 388 (57 %) | 1248 (60 %) |  |
| **Energy drink consumption:** |  |  |  |  | < 0.001 |
| High | 2 (0 %) | 1 (0 %) | 1 (0 %) | 4 (0 %) |  |
| Low | 636 (91 %) | 673 (97 %) | 672 (98 %) | 1981 (95 %) |  |
| Moderate | 58 (8 %) | 21 (3 %) | 11 (2 %) | 90 (4 %) |  |
| **Substance use (past month):** |  |  |  |  | 0.004 |
| No | 631 (91 %) | 639 (92 %) | 651 (95 %) | 1921 (93 %) |  |
| Yes | 65 (9 %) | 56 (8 %) | 33 (5 %) | 154 (7 %) |  |
| **Emotional well-being (GHQ-12):** |  |  |  |  | 0.004 |
| Good | 77 (11 %) | 99 (14 %) | 110 (16 %) | 286 (14 %) |  |
| Moderate | 307 (44 %) | 331 (48 %) | 328 (48 %) | 966 (47 %) |  |
| Poor | 312 (45 %) | 265 (38 %) | 246 (36 %) | 823 (40 %) |  |
| **Satisfaction with relationships:** |  |  |  |  | < 0.001 |
| High | 441 (63 %) | 512 (74 %) | 523 (76 %) | 1476 (71 %) |  |
| Low | 23 (3 %) | 18 (3 %) | 12 (2 %) | 53 (3 %) |  |
| Medium | 229 (33 %) | 165 (24 %) | 147 (21 %) | 541 (26 %) |  |
| Missing | 3 (0.4%) | 0 (0%) | 2 (0.3%) | 5 (0.2%) |  |

**Note:** Tertiles were computed separately based on the HEASUS score distribution within the students population group. Values are presented as mean (SD) for continuous variables and n (%) for categorical variables. *p value overall* corresponds to global p-values obtained from comparison tests across students tertiles. *Chronic mental health condition*: binary indicator for having any of the mental conditions (stress, anxiety, depression); *Migraine (chronic):* binary indicator of self-reported diagnosis of migraine (yes/no) categorized as part of chronic conditions; *Any disability* (including one of the following: vision, hearing, mobility, physical function, daily activities, assistive devices); *Meals with others:* High (≥5/week), Medium (2–4/week), and Low (<2/week) frequency of shared meals (breakfast, lunch, dinner); *Binge drinking:* Never, Occasional (monthly or less), and Frequent (weekly or more) for consuming ≥ 6 alcoholic units in one occasion; *Coffee and energy drink consumption:* Low (≤1 time/week), Moderate (2–6 times/week or once/day), and High (≥2 times/day); *Satisfaction with relationships:* Low (dissatisfied), Medium (acceptable), and High (satisfied) based on satisfaction with partner, family, or friends (5-point scale). Missing values in variables such as *any disability*, *satisfaction with relationships* and *binge drinking* reflected non-response (in one or more of the underlying questions), “prefer not to answer” or “not applicable” options. **Test**: Kruskal–Wallis (K-W) test used for continuous variables and Chi-square (χ2) for categorical variables.

**B. Staff**

|  | **T1**  **(N=182)** | **T2**  **(N=174)** | **T3**  **(N=177)** | **[ALL]**  **(N=533)** | ***p value overall*** |
| --- | --- | --- | --- | --- | --- |
| **HEASUS index range** | ≤ 5.93 | 5.94 - 6.70 | ≥ 6.71 | **-** | **-** |
| **HEASUS score (range -1 to 10)** | 5.3 (± 0.61) | 6.3 (± 0.21) | 7.3 (± 0.46) | 6.3 (± 0.95) | - |
| **Gender:** |  |  |  |  | < 0.001 |
| Male | 74 (41 %) | 52 (30 %) | 33 (19 %) | 159 (30 %) |  |
| Female | 105 (58 %) | 122 (70 %) | 143 (81 %) | 370 (69 %) |  |
| Others | 3 (2 %) | 0 (0 %) | 1 (1 %) | 4 (1 %) |  |
| **Age** | 44 (± 8.4) | 45 (± 9.4) | 45 (± 9.7) | 45 (± 9.2) | 0.545 |
| **Body Mass Index (BMI)** | 24 (± 3.9) | 24 (± 3.6) | 23 (± 3.6) | 24 (± 3.7) | 0.461 |
| **Work type:** |  |  |  |  | < 0.001 |
| PAS | 127 (70 %) | 104 (60 %) | 74 (42 %) | 305 (57 %) |  |
| PDI | 28 (15 %) | 40 (23 %) | 48 (27 %) | 116 (22 %) |  |
| PDC | 27 (15 %) | 30 (17 %) | 55 (31 %) | 112 (21 %) |  |
| **Caregiving status:** |  |  |  |  | 0.63 |
| None | 86 (47 %) | 82 (47 %) | 88 (50 %) | 256 (48 %) |  |
| Children only | 86 (47 %) | 83 (48 %) | 73 (41 %) | 242 (45 %) |  |
| Dependents only | 6 (3 %) | 4 (2 %) | 7 (4 %) | 17 (3 %) |  |
| Children and dependents | 4 (2 %) | 5 (3 %) | 9 (5 %) | 18 (3 %) |  |
| **Birth place:** |  |  |  |  | 0.151 |
| Catalonia | 141 (77 %) | 134 (77 %) | 123 (69 %) | 398 (75 %) |  |
| Spain or Other | 41 (23 %) | 40 (23 %) | 54 (31 %) | 135 (25 %) |  |
| **Self-perceived socioeconomic status:** |  |  |  |  | 0.285 |
| Worse | 15 (8 %) | 8 (5 %) | 6 (3 %) | 29 (5 %) |  |
| Average | 117 (64 %) | 110 (63 %) | 116 (66 %) | 343 (64 %) |  |
| Better | 50 (27 %) | 56 (32 %) | 55 (31 %) | 161 (30 %) |  |
| **Education level:** |  |  |  |  | 0.02 |
| Low | 7 (4 %) | 5 (3 %) | 4 (2 %) | 16 (3 %) |  |
| Medium | 29 (16 %) | 14 (8 %) | 12 (7 %) | 55 (10 %) |  |
| High | 146 (80 %) | 155 (89 %) | 161 (91 %) | 462 (87 %) |  |
| **Occupational physical activity:** |  |  |  |  | 0.019 |
| Low | 152 (84 %) | 140 (80 %) | 120 (68 %) | 412 (77 %) |  |
| Moderate | 26 (14 %) | 28 (16 %) | 44 (25 %) | 98 (18 %) |  |
| High | 2 (1 %) | 3 (2 %) | 6 (3 %) | 11 (2 %) |  |
| Missing | 2 (1.1%) | 3 (1.7%) | 7 (4.0%) | 12 (2.3%) |  |
| **Self-perceived health:** |  |  |  |  | < 0.001 |
| Regular | 22 (12 %) | 9 (5 %) | 8 (5 %) | 39 (7 %) |  |
| Good | 91 (50 %) | 89 (51 %) | 64 (36 %) | 244 (46 %) |  |
| Very Good | 69 (38 %) | 76 (44 %) | 105 (59 %) | 250 (47 %) |  |
| **Sleeping hours per day** | 7.1 (± 0.88) | 7.3 (± 0.76) | 7.2 (± 0.83) | 7.2 (± 0.83) | 0.46 |
| **Sleep quality:** |  |  |  |  | 0.341 |
| Regular or Bad | 93 (51 %) | 91 (52 %) | 86 (49 %) | 270 (51 %) |  |
| Good | 13 (7 %) | 16 (9 %) | 24 (14 %) | 53 (10 %) |  |
| Very Good | 76 (42 %) | 67 (39 %) | 67 (38 %) | 210 (39 %) |  |
| **Chronic mental health:** |  |  |  |  | 0.358 |
| No | 159 (87 %) | 152 (87 %) | 162 (92 %) | 473 (89 %) |  |
| Yes | 23 (13 %) | 22 (13 %) | 15 (8 %) | 60 (11 %) |  |
| **Migraine (chronic):** |  |  |  |  | 0.647 |
| No | 165 (91 %) | 161 (93 %) | 165 (93 %) | 491 (92 %) |  |
| Yes | 17 (9 %) | 13 (7 %) | 12 (7 %) | 42 (8 %) |  |
| **Any disability:** |  |  |  |  | 0.159 |
| No | 172 (95 %) | 163 (94 %) | 172 (97 %) | 507 (95 %) |  |
| Yes | 8 (4 %) | 7 (4 %) | 2 (1 %) | 17 (3 %) |  |
| Missing | 2 (1.1%) | 4 (2.3%) | 3 (1.7%) | 9 (1.7%) |  |
| **Plant Rich Diet (PRD):** |  |  |  |  | < 0.001 |
| No | 63 (35 %) | 40 (23 %) | 13 (7 %) | 116 (22 %) |  |
| Yes | 119 (65 %) | 134 (77 %) | 164 (93 %) | 417 (78 %) |  |
| **Western Diet (WD):** |  |  |  |  | < 0.001 |
| No | 156 (86 %) | 168 (97 %) | 173 (98 %) | 497 (93 %) |  |
| Yes | 26 (14 %) | 6 (3 %) | 4 (2 %) | 36 (7 %) |  |
| **Self-perceived healthy eating habits (range 0 to 10)** | 6.4 (± 1.8) | 7.4 (± 1.4) | 7.8 (± 1.6) | 7.2 (± 1.7) | < 0.001 |
| **Meal context:** |  |  |  |  | 0.205 |
| Alone | 20 (11 %) | 17 (10 %) | 29 (16 %) | 66 (12 %) |  |
| Distracted | 85 (47 %) | 77 (44 %) | 66 (37 %) | 228 (43 %) |  |
| Social | 77 (42 %) | 80 (46 %) | 82 (46 %) | 239 (45 %) |  |
| **Meals with others:** |  |  |  |  | 0.343 |
| High | 68 (37 %) | 75 (43 %) | 84 (47 %) | 227 (43 %) |  |
| Low | 23 (13 %) | 16 (9 %) | 15 (8 %) | 54 (10 %) |  |
| Medium | 90 (49 %) | 83 (48 %) | 78 (44 %) | 251 (47 %) |  |
| Missing | 1 (0.5%) | 0 (0%) | 0 (0%) | 1 (0.2%) |  |
| **Physical Activity (PA) level:** |  |  |  |  | 0.105 |
| Low | 36 (20 %) | 24 (14 %) | 17 (10 %) | 77 (14 %) |  |
| Moderate | 123 (68 %) | 127 (73 %) | 136 (77 %) | 386 (72 %) |  |
| High | 23 (13 %) | 23 (13 %) | 24 (14 %) | 70 (13 %) |  |
| **Sitting hours per day** | 8.3 (± 2.8) | 8.1 (± 2.9) | 7.9 (± 2.7) | 8.1 (± 2.8) | 0.044 |
| **Sedentary behavior (SB):** |  |  |  |  | 0.216 |
| Active | 89 (49 %) | 82 (47 %) | 99 (56 %) | 270 (51 %) |  |
| Sedentary | 93 (51 %) | 92 (53 %) | 78 (44 %) | 263 (49 %) |  |
| **Transport mode:** |  |  |  |  | < 0.001 |
| Walk | 58 (32 %) | 61 (35 %) | 71 (40 %) | 190 (36 %) |  |
| Public transport | 54 (30 %) | 43 (25 %) | 31 (18 %) | 128 (24 %) |  |
| Car or Motorbike | 64 (35 %) | 55 (32 %) | 47 (27 %) | 166 (31 %) |  |
| Bike & Others | 6 (3 %) | 15 (9 %) | 28 (16 %) | 49 (9 %) |  |
| **Alcohol consumption:** |  |  |  |  | 0.205 |
| Never | 19 (10 %) | 28 (16 %) | 29 (16 %) | 76 (14 %) |  |
| Almost Never | 28 (15 %) | 33 (19 %) | 31 (18 %) | 92 (17 %) |  |
| Occasionally | 68 (37 %) | 70 (40 %) | 68 (38 %) | 206 (39 %) |  |
| Weekly | 67 (37 %) | 43 (25 %) | 49 (28 %) | 159 (30 %) |  |
| **Binge drinking:** |  |  |  |  | 0.732 |
| Frequent | 1 (1 %) | 1 (1 %) | 0 (0 %) | 2 (0 %) |  |
| Never | 126 (69 %) | 117 (67 %) | 112 (63 %) | 355 (67 %) |  |
| Occasional | 36 (20 %) | 28 (16 %) | 36 (20 %) | 100 (19 %) |  |
| Missing | 19 (10.4%) | 28 (16.1%) | 29 (16.4%) | 76 (14.3%) |  |
| **Alcohol quantity:** |  |  |  |  | 0.208 |
| ≥5 drinks | 5 (3 %) | 0 (0 %) | 2 (1 %) | 7 (1 %) |  |
| 1–2 drinks | 133 (73 %) | 127 (73 %) | 129 (73 %) | 389 (73 %) |  |
| 3-4 drinks | 25 (14 %) | 19 (11 %) | 17 (10 %) | 61 (11 %) |  |
| Missing | 19 (10.4%) | 28 (16.1%) | 29 (16.4%) | 76 (14.3%) |  |
| **Smoking status:** |  |  |  |  | 0.011 |
| Non-smoker | 129 (71 %) | 143 (82 %) | 150 (85 %) | 422 (79 %) |  |
| Occasional smoker | 23 (13 %) | 13 (7 %) | 15 (8 %) | 51 (10 %) |  |
| Regular smoker | 30 (16 %) | 18 (10 %) | 12 (7 %) | 60 (11 %) |  |
| **Coffee consumption:** |  |  |  |  | 0.043 |
| High | 29 (16 %) | 38 (22 %) | 51 (29 %) | 118 (22 %) |  |
| Low | 27 (15 %) | 30 (17 %) | 22 (12 %) | 79 (15 %) |  |
| Moderate | 126 (69 %) | 106 (61 %) | 104 (59 %) | 336 (63 %) |  |
| **Energy drink consumption:** |  |  |  |  | 0.014 |
| High | 1 (1 %) | 0 (0 %) | 0 (0 %) | 1 (0 %) |  |
| Low | 178 (98 %) | 173 (99 %) | 176 (99 %) | 527 (99 %) |  |
| Moderate | 3 (2 %) | 1 (1 %) | 1 (1 %) | 5 (1 %) |  |
| **Substance use (past month):** |  |  |  |  | 0.107 |
| No | 168 (92 %) | 169 (97 %) | 169 (95 %) | 506 (95 %) |  |
| Yes | 14 (8 %) | 5 (3 %) | 8 (5 %) | 27 (5 %) |  |
| **Emotional well-being (GHQ-12):** |  |  |  |  | 0.8 |
| Good | 15 (8 %) | 18 (10 %) | 18 (10 %) | 51 (10 %) |  |
| Moderate | 116 (64 %) | 107 (61 %) | 117 (66 %) | 340 (64 %) |  |
| Poor | 51 (28 %) | 49 (28 %) | 42 (24 %) | 142 (27 %) |  |
| **Satisfaction with relationships:** |  |  |  |  | 0.976 |
| High | 153 (84 %) | 147 (84 %) | 149 (84 %) | 449 (84 %) |  |
| Low | 1 (1 %) | 2 (1 %) | 2 (1 %) | 5 (1 %) |  |
| Medium | 28 (15 %) | 25 (14 %) | 25 (14 %) | 78 (15 %) |  |
| Missing | 0 (0%) | 0 (0%) | 1 (0.6%) | 1 (0.2%) |  |

**Note:** Tertiles were computed separately based on the HEASUS score distribution within the staff population group. Values are presented as mean (SD) for continuous variables and n (%) for categorical variables. *p value overall* corresponds to global p-values obtained from comparison tests across staff tertiles. *Work type*: PAS (Administrative and Service Staff), PDI (Teaching and Research Staff), and PDC (Administrative and Service Staff). *Education level*: Low (Secondary or High School, including “Other”), Medium (Vocational training or first university cycle), and High (Second university cycle/postgraduate education); *Occupational PA*: Low (mostly sitting or standing), Moderate (frequent walking), and High (physically demanding activity); *Chronic mental health condition*: binary indicator for having any of the mental conditions (stress, anxiety, depression); *Migraine (chronic):* binary indicator of self-reported diagnosis of migraine (yes/no) categorized as part of chronic conditions; *Any disability* (including one of the following: vision, hearing, mobility, physical function, daily activities, assistive devices); *Meals with others:* High (≥5/week), Medium (2–4/week), and Low (<2/week) frequency of shared meals (breakfast, lunch, dinner); *Binge drinking:* Never, Occasional (monthly or less), and Frequent (weekly or more) for consuming ≥ 6 alcoholic units in one occasion; *Coffee and energy drink consumption*: Low (≤1 time/week), Moderate (2–6 times/week or once/day), and High (≥2 times/day); *Satisfaction with relationships:* Low (dissatisfied), Medium (acceptable), and High (satisfied) based on satisfaction with partner, family, or friends (5-point scale). Missing values in variables such as *occupational physical activity*, *any disability, meals with others, alcohol quantity* and *binge drinking* reflected non-response (in one or more of the underlying questions), “prefer not to answer” or “not applicable” options. **Test**: Kruskal–Wallis (K-W) test used for continuous variables and Chi-square (χ2) for categorical variables.

**Supplementary Table 4: Adjusted quantile regression coefficients (τ = 0.25, 0.55, 0.75) for the HEASUS Index by predictor variables.**

1. **Students**

| **Category** | **Variable** | **τ = 0.25** | **0.25 SE** | **τ = 0.55** | **0.55 SE** | **τ = 0.75** | **0.75 SE** |
| --- | --- | --- | --- | --- | --- | --- | --- |
|  | (Intercept) | 4.231^***^ | 0.239 | 5.292^***^ | 0.197 | 5.674^***^ | 0.226 |
| **Dietary habits** | Plant Rich Diet = Yes | 0.774^***^ | 0.087 | 0.692^***^ | 0.082 | 0.699^***^ | 0.077 |
|  | Western Diet = Yes | -0.533^***^ | 0.103 | -0.591^***^ | 0.089 | -0.626^***^ | 0.104 |
|  | Coffee consumption = Low | -0.362^**^ | 0.108 | -0.446^***^ | 0.092 | -0.250^*^ | 0.103 |
|  | Coffee consumption = Moderate | -0.194^*^ | 0.094 | -0.258^***^ | 0.068 | -0.256^***^ | 0.07 |
| **Demographics** | Age | 0.023^***^ | 0.003 | 0.019^***^ | 0.003 | 0.020^***^ | 0.003 |
|  | Gender = Male | -0.262^**^ | 0.091 | -0.214^**^ | 0.068 | -0.276^***^ | 0.068 |
|  | BMI = Overweight | -0.260^***^ | 0.077 | -0.229^***^ | 0.066 | -0.155^*^ | 0.073 |
|  | BMI = Underweight | 0.019 | 0.149 | -0.113 | 0.126 | 0.038 | 0.127 |
|  | Type of Study = Health | 0.18 | 0.147 | 0.291^**^ | 0.107 | 0.366^**^ | 0.113 |
|  | Type of Study = Social/Legal | 0.009 | 0.084 | 0.007 | 0.073 | -0.009 | 0.08 |
|  | Type of Study = Engineering | 0.034 | 0.113 | 0.036 | 0.1 | 0.13 | 0.11 |
|  | Student type = Master/postgraduate | 0.178^*^ | 0.08 | 0.163^*^ | 0.063 | 0.159^*^ | 0.065 |
|  | Student type = PhD | -0.074 | 0.24 | 0.197 | 0.222 | 0.466^*^ | 0.214 |
| **Physical activity and sedentarism** | Physical Activity level = High | 0.479^***^ | 0.108 | 0.527^***^ | 0.104 | 0.617^***^ | 0.102 |
|  | Physical Activity level = Moderate | 0.290^***^ | 0.083 | 0.350^***^ | 0.083 | 0.373^***^ | 0.079 |
|  | Sedentary behavior = Sedentary | 0.024 | 0.075 | -0.067 | 0.061 | -0.031 | 0.073 |
|  | Transport Mode = Bike & Others | 0.218 | 0.152 | 0.126 | 0.131 | 0.07 | 0.154 |
|  | Transport Mode = Car or Motorbike | -0.227^*^ | 0.088 | -0.134^*^ | 0.065 | -0.139 | 0.075 |
|  | Transport Mode = Public Transport | -0.213 | 0.113 | -0.092 | 0.081 | -0.139 | 0.088 |
| **Mental and emotional well being** | GHQ12 = Moderate | -0.116 | 0.102 | -0.162^*^ | 0.078 | -0.134 | 0.093 |
|  | GHQ12 = Poor | -0.096 | 0.12 | -0.126 | 0.087 | -0.065 | 0.104 |
|  | Quality Sleep = Good | -0.127 | 0.087 | -0.085 | 0.066 | -0.144 | 0.078 |
|  | Quality Sleep = Very Good | 0.033 | 0.093 | 0.027 | 0.072 | -0.024 | 0.079 |
|  | Meal Context = Distracted | 0.063 | 0.115 | 0.073 | 0.078 | 0.039 | 0.088 |
|  | Meal Context = Social | 0.145 | 0.078 | 0.038 | 0.06 | -0.025 | 0.069 |
| **Substances consumption** | Smoking Status = Occasional smoker | -0.046 | 0.104 | -0.05 | 0.097 | 0.03 | 0.099 |
|  | Smoking Status = Regular smoker | -0.323^*^ | 0.126 | -0.242^*^ | 0.098 | -0.183^*^ | 0.078 |
|  | Substance Use (past month) = Yes | -0.127 | 0.123 | -0.290^**^ | 0.095 | -0.338^**^ | 0.123 |

BMI: Body Mass Index; GHQ12: General Health Questionnaire-12 item; τ: Tau value at which the coefficient is calculated; SE: Standard error of the coefficient. Significance levels indicated by stars: ^***^p < 0.001; ^**^p < 0.01; ^*^p < 0.05.

**B. Staff**

| **Category** | **Variable** | **τ = 0.25** | **0.25 SE** | **τ = 0.55** | **0.55 SE** | **τ = 0.75** | **0.75 SE** |
| --- | --- | --- | --- | --- | --- | --- | --- |
|  | (Intercept) | 4.962^***^ | 0.708 | 0.619 | 5.809^***^ | 6.633^***^ | 0.529 |
| **Dietary habits** | Plant Rich Diet = Yes | 0.423^**^ | 0.142 | 0.121 | 0.418^***^ | 0.405^**^ | 0.129 |
|  | Western Diet = Yes | -0.678^**^ | 0.23 | 0.199 | -0.642^**^ | -0.574^*^ | 0.244 |
|  | Coffee consumption = Low | -0.002 | 0.192 | 0.158 | -0.288 | -0.282 | 0.192 |
|  | Coffee consumption = Moderate | -0.064 | 0.159 | 0.135 | -0.249 | -0.265^*^ | 0.134 |
| **Demographics** | Age | 0.011 | 0.008 | 0.005 | 0.009 | 0.006 | 0.006 |
|  | Gender = Male | -0.324^*^ | 0.141 | 0.107 | -0.296^**^ | -0.422^***^ | 0.116 |
|  | BMI = Overweight | -0.112 | 0.132 | 0.109 | -0.008 | 0.052 | 0.116 |
|  | BMI = Underweight | -0.074 | 0.338 | 0.29 | -0.146 | 0.072 | 0.355 |
|  | Work Type = PDI | 0.406^*^ | 0.167 | 0.128 | 0.393^**^ | 0.23 | 0.13 |
|  | Work Type = PDC | 0.174 | 0.162 | 0.132 | 0.209 | 0.214 | 0.176 |
|  | Education Level = Medium | -0.135 | 0.484 | 0.429 | -0.148 | -0.454 | 0.309 |
|  | Education Level = High | 0.175 | 0.47 | 0.401 | 0.178 | -0.07 | 0.26 |
|  | Self-perceived health = Good | 0.213 | 0.228 | 0.194 | -0.002 | 0.116 | 0.195 |
|  | Self-perceived health = Very Good | 0.252 | 0.255 | 0.201 | 0.258 | 0.319 | 0.22 |
| **Physical activity and sedentarism** | Physical Activity level = High | 0.057 | 0.241 | 0.189 | 0.131 | -0.014 | 0.228 |
|  | Physical Activity level = Moderate | 0.115 | 0.152 | 0.151 | 0.214 | 0.027 | 0.157 |
|  | Sedentary behavior = Sedentary | 0.165 | 0.111 | 0.091 | 0.035 | -0.074 | 0.104 |
|  | Transport Mode = Bike & Others | 0.438^*^ | 0.205 | 0.157 | 0.141 | 0.136 | 0.173 |
|  | Transport Mode = Car or Motorbike | -0.221 | 0.14 | 0.11 | -0.248^*^ | -0.290^*^ | 0.134 |
|  | Transport Mode = Public Transport | -0.098 | 0.183 | 0.126 | -0.083 | -0.055 | 0.136 |
|  | Occupational PA = Low | 0.189 | 0.195 | 0.213 | 0.32 | 0.029 | 0.187 |
|  | Occupational PA = Moderate | 0.116 | 0.152 | 0.156 | 0.147 | -0.018 | 0.152 |
| **Mental and emotional well being** | GHQ12 = Moderate | -0.268 | 0.213 | 0.148 | -0.095 | -0.116 | 0.172 |
|  | GHQ12 = Poor | -0.174 | 0.234 | 0.166 | -0.077 | -0.023 | 0.192 |
|  | Quality Sleep = Good | 0.188 | 0.262 | 0.201 | 0.191 | 0.371 | 0.212 |
|  | Quality Sleep = Very Good | -0.077 | 0.129 | 0.102 | -0.08 | -0.031 | 0.112 |
| **Substances consumption** | Smoking Status = Occasional Smoker | -0.189 | 0.216 | 0.179 | -0.093 | -0.047 | 0.201 |
|  | Smoking Status = Regular Smoker | -0.3 | 0.235 | 0.143 | -0.125 | -0.27 | 0.165 |

BMI: Body Mass Index; Work type: PAS (Administrative and Service Staff), PDI (Teaching and Research Staff), and PDC (Administrative and Service Staff); Education level: Low (Secondary or High School, including “Other”), Medium (Vocational training or first university cycle), and High (Second university cycle/postgraduate education); Occupational physical activity: Low (mostly sitting or standing), Moderate (frequent walking), and High (physically demanding activity); GHQ12: General Health Questionnaire-12 item; τ = Tau value at which the coefficient is calculated; SE = Standard error of the coefficient. Significance levels indicated by stars: ^***^p < 0.001; ^**^p < 0.01; ^*^p < 0.05.
